# Attendance and compliance with an exercise program during localized breast cancer treatment in a randomized controlled trial: The PACT study

**DOI:** 10.1371/journal.pone.0215517

**Published:** 2019-05-08

**Authors:** Lenja Witlox, Miranda J. Velthuis, Jennifer H. Boer, Charlotte N. Steins Bisschop, Elsken van der Wall, Wout J. T. M. van der Meulen, Carin D. Schröder, Petra H. M. Peeters, Anne M. May

**Affiliations:** 1 Julius Center for Health Sciences and Primary Care, University Medical Center, Utrecht University, Utrecht, the Netherlands; 2 Netherlands Comprehensive Cancer Organisation, Utrecht, the Netherlands; 3 Department of Medical Oncology, University Medical Center Utrecht, Utrecht University, Utrecht, The Netherlands; 4 Brain Center Rudolf Magnus and Center of Excellence for Rehabilitation Medicine, University Medical Center Utrecht, Utrecht University, Utrecht, the Netherlands; 5 De Hoogstraat Rehabilitation, Utrecht, the Netherlands; 6 School of Public Health, Imperial College London, London, United Kingdom; Istituto di Ricovero e Cura a Carattere Scientifico Centro di Riferimento Oncologico della Basilicata, ITALY

## Abstract

**Purpose:**

Maintaining high adherence rates (session attendance and compliance) in exercise programs during breast cancer treatment can be challenging. We aimed to identify adherence rates and predictors to an exercise program during adjuvant breast cancer treatment.

**Methods:**

Ninety-two patients with localized breast cancer undergoing chemotherapy were randomly assigned to an 18-week supervised moderate-to-high intensity aerobic and resistance exercise program, including two 1-hour sessions/week. Additionally, participants were asked to be physically active for at least 30 minutes/day on at least three other days. We report median percentages for attendance, compliance with the prescribed duration and intensity of aerobic and muscle strength exercises, and the exercise advice given. Predictors included in univariate and multivariable linear regression models were demographical, tumor- and treatment-related factors, constructs of the theory of planned behavior, psychological and physical factors.

**Results:**

Patients attended 83% (interquartile range: 69–91%) of the supervised sessions. Compliance with the duration of aerobic exercise, high-intensity aerobic exercise (cycling at the ventilatory threshold), muscle strength exercises and the exercise advice were 88%(64–97%), 50%(22–82%), 84%(65–94%) and 61%(33%–79%), respectively. Education, radiotherapy, BMI and physical fatigue were important predictors of adherence to supervised exercise. Beliefs about planned behaviors were important predictors, especially for compliance with the exercise advice.

**Conclusions:**

Attendance to and compliance with an 18-week aerobic and strength exercise program were high. The lowest compliance was found for high-intensity supervised aerobic exercise. The identified predictors should be considered when designing or adapting exercise programs for patients with localized breast cancer to increase adherence.

**Trial registration:**

Current Controlled Trials ISRCTN43801571

Dutch Trial Register NTR2138

## Background

In our randomized Physical Activity during Cancer Treatment (PACT) study, we showed that an 18-week exercise program had beneficial effects on fatigue and physical fitness in newly diagnosed breast cancer patients undergoing adjuvant treatment [1]. Several meta-analyses have also reported that supervised exercise interventions during cancer treatment have positive effects on cancer-related fatigue, physical functioning and quality of life [2–6]; however, in general the reported effect sizes were small [1, 7].

An important component facilitating the optimal effectiveness of exercise programs is a high level of exercise adherence, which is defined by the World Health Organization as “the extent to which a person’s behavior corresponds with agreed recommendations” [8]. This definition of adherence incorporates both the number of sessions attended as well as the compliance with the prescribed intensity and duration of the individual training sessions. Adherence to prescribed exercise interventions during cancer treatment is known to be challenging, particularly because of treatment-related barriers [9, 10]. According to a recent review of Neil-Sztramko et al. (2017), studies did not include all components of exercise adherence rates such as attendance and compliance with the prescribed intensity and duration [11]. In previous studies with varying frequencies, durations, and timings of exercise programs among breast cancer patients during treatment, attendance rates for supervised exercise sessions were between 70**–**79% [12–17]. Little is known about the compliance of breast cancer patients with prescribed programs. Breast cancer patients undergoing treatment who followed a home-based moderate-intensity walking program had a compliance rate of 87% for intensity and 98% for the duration of the program [18]. However, the compliance rates during supervised exercise programs including high-intensity training have not been reported; and so far, it is unknown whether patients comply to the high intensities. Also, compliance with resistance exercises is currently unknown in breast cancer patients participating in exercise trials.

Identifying the factors that predict adherence rates during supervised exercise could lead to improved strategies for enhancing adherence to future interventions, thereby enhancing their effectiveness. It is important to distinguish between trials utilizing exercise interventions during or after cancer treatment because the factors predicting adherence may differ. For example, treatment-related factors may be less important after the cancer treatment has finished. In addition, predictors of adherence may also differ between home-based and supervised exercise. The systematic review of Kampshoff et al. stated that exercise history was a prominent predictor of adherence in cancer patients during or after treatment (19), which is in accordance with a recent study [19, 20]. A growing number of studies have reported predictors of exercise attendance or compliance especially during adjuvant breast cancer treatment [12, 13, 18], three of which applied supervised programs [12, 13].Two of these studies found that a high peak oxygen consumption (VO_2peak_), a measure of physical fitness, was an important predictor of high attendance rates [12, 13]. Furthermore, quality of life, being employed before treatment and a high personal income predicted high attendance rates in breast cancer patients [21]. However, in these studies predictors of exercise compliance were not addressed. Two studies investigated predictors of compliance, but both applied a home-based program. One study showed that patients who were less fatigued had a higher compliance with both walking duration and intensity [18]. Patients who perceived a higher importance of exercise, who had an early stage of disease, and who were employed had a higher compliance with exercise intensity [18]. The study of Nyrop et al. reported that being Caucasian or reporting higher walking minutes prior to the start of chemotherapy was associated with greater walking steps per week in a home-based exercise intervention [22]. To our knowledge, no studies were performed reporting predictors about compliance in supervised exercise programs during treatment in breast cancer patients. The review of Kampshoff et al. recommended that future studies should specifically focus on both the predictors of attendance and different parts of compliance [19]. Since self-efficacy about participation during the exercise sessions was found as predictor for exercise adherence in cancer survivors [23], this might also be of interest in cancer patient during treatment. Therefore, it is recommended to get insight in behavioral constructs to increase our understanding of exercise behavior in cancer patients. A commonly used framework to understand exercise behavior in cancer patients is Ajzen’s theory of planned behavior which links beliefs and behavior [24]. The patients’ beliefs regarding exercise might influence their adherence to the exercise program and should therefore be assessed when looking for possible factors predicting adherence to physical exercise.

The aim of the present study is to identify adherence rates (both attendance and compliance rates) of patients taking part in an 18-week supervised aerobic and strength exercise intervention and exercise advice during adjuvant breast cancer treatment. Other aims included exploring which demographical, tumor-related, treatment-related, psychological, and physical factors, as well as which constructs of the theory of planned behavior, predicted attendance and compliance.

## Methods

### Setting and patients

The design of the PACT study (ISRCTN43801571) has been described in detail elsewhere [1, 25, 26]. In short, breast (n = 202) and colon cancer (n = 33) patients were included in the study after written informed consent was signed. A concealed computer-generated randomization, following a 1:1 ratio was used to allocate participants in the exercise intervention group or the control group. Randomization was stratified by age, adjuvant treatment (radiotherapy yes/no before chemotherapy), use of tissue expander and hospital. Sequential balancing was used to allocate participants to the study groups. In the present exploratory analysis, only breast cancer patients who were randomly allocated to the exercise intervention were included (n = 102).

**Fig 1:**
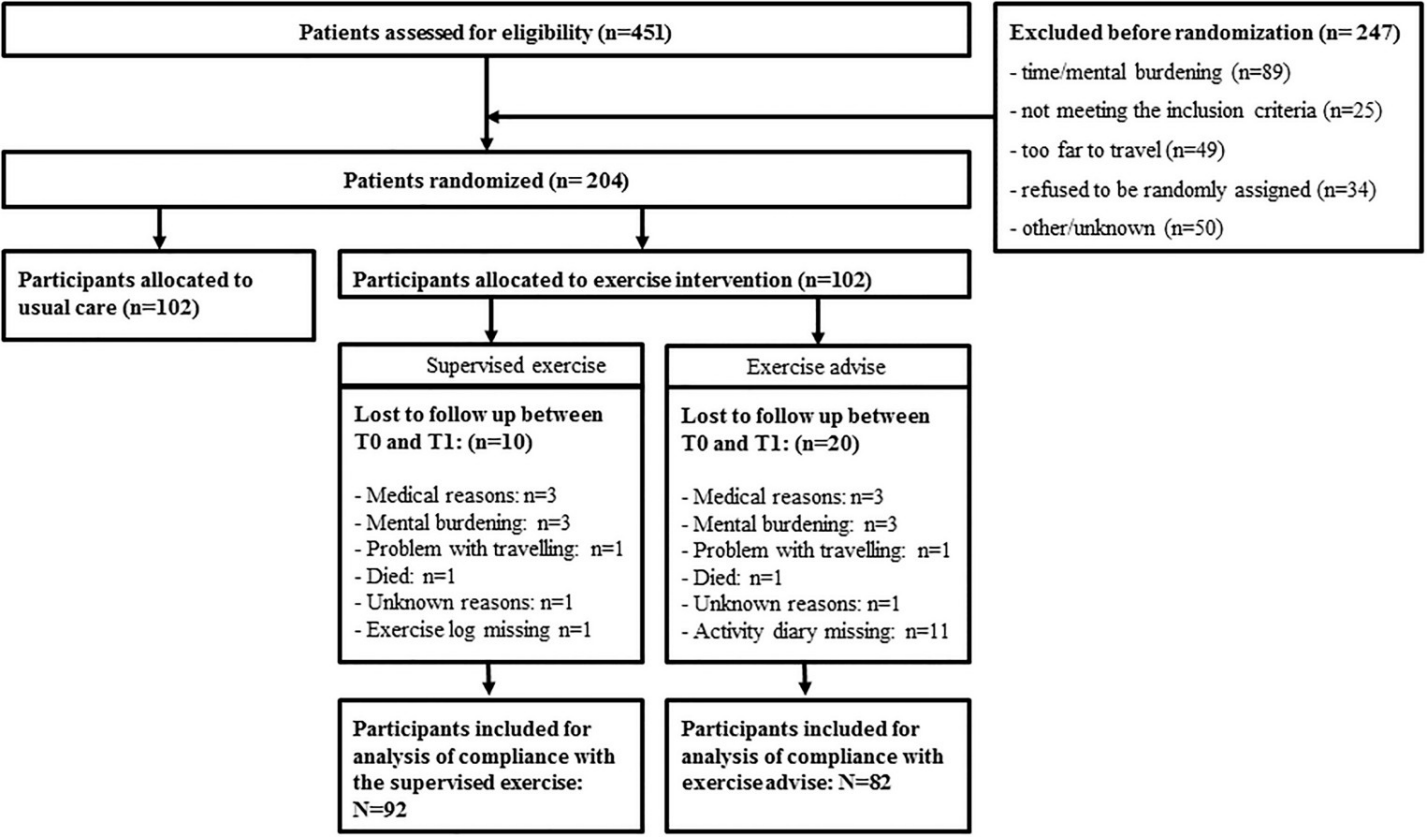
Flow of the participants through the study.

Since 10 exercise logs were not returned, attendance to and compliance with the supervised exercise program was determined for 92 breast cancer patients. Compliance with the exercise advice was determined for 82 patients, due to missing activity diaries (see Fig 1 for the flow chart). Since the original study was not primarily powered for the current analysis, the results should be interpreted as exploratory data. The sample size of the original study was calculated to detect an intervention effect on fatigue of 2 units improvement in the Multiple fatigue Inventory (MFI) questionnaire [25]. Patients were recruited in seven hospitals in the Netherlands between 2010 and 2012. Inclusion criteria were: histological diagnosis of cancer less than six weeks before study recruitment (ten weeks for patients with mastectomy and immediate reconstruction using a tissue expander); stage M0; scheduled for chemotherapy; age 25–75 years; not treated for any other cancer in the five years preceding recruitment (except basal skin cancer); able to read and understand the Dutch language; Karnovsky Performance Status of 60 or higher; able to walk 100 meters or more; no contra-indications for physical activity. The Medical Ethics Committee of the University Medical Centre Utrecht and the local Ethical Boards of the participating hospitals approved the study (07-271/O) and the local Ethical Boards of the participating hospitals (i.e., St. Antonius Hospital, Nieuwegein and Woerden; Diakonessenhuis Hospital, Utrecht; Meander Medical Centre, Amersfoort; Rivierenland Hospital, Tiel; Orbis Medical Centre, Sittard).

### Exercise intervention

Patients in the intervention group were offered an 18-week supervised exercise program, in addition to the usual care. The program included two 1-hour sessions per week of combined aerobic and muscle strength exercises supervised by a physiotherapist. In addition, patients were asked to be physically active for at least 30 minutes per day on at least three other days [27]. Several principles of Bandura’s social cognitive theory were incorporated in the exercise program in order to motivate the patients to adhere to the exercise program and to maintain high physical activity levels during and after the supervised exercise program [28].

#### Supervised exercise program

The exercise program was individualized to the patients’ preferences and fitness levels. Aerobic fitness and muscle strength were determined, respectively, by means of a cardiopulmonary exercise test (CPET) and a one-repetition maximum (1-RM) test at the baseline [29]. The aerobic training included interval training of alternating intensities at or below the ventilatory threshold (VT), as monitored by the heart rate. The exercises were performed on cardiovascular fitness equipment of choice. Muscle strength training was performed for all major muscle groups: arms, legs, shoulders, and trunk. In order to minimize the injury risk of strength assessment, 1-RM strength was predicted by using regression equation prescribed by the American Society of Exercise Physiologists (ASEP)[30]. During all muscle strength assessments, the weight will be estimated that can be lifted between 4 and 10 repetitions. See S1 Table for details. Training intensity was re-assessed every four weeks. The weight used for the muscle strength exercises was adjusted every four weeks based on the estimated 1-RM from a submaximal muscle strength test. If necessary, weight was adjusted between the scheduled tests, although the participant was primarily asked to complete the total repetitions of each exercise prior to adding weight resistance.

The physiotherapists were instructed to closely follow the exercise protocol; however, if patients were not feeling well, adjustments were allowed and documented. If adjustments were necessary, we instructed the physiotherapist to reduce the exercise intensity prior to a reduction of the duration.

#### Exercise advice

Patients were asked to be physically active at moderate intensity for at least 30 minutes for a total of five days a week, i.e. at least three other days of the week in addition to the two supervised PACT exercise days. Patients were encouraged to embed this activity in their daily lifestyle, in order to make it a habit.

#### The behavioral component

Bandura’s social cognitive theory emphasizes the role of cognitive processes in behavior [28]. An important construct in the theory is self-efficacy, defined as an individual’s own beliefs in his/her capacity to organize and execute the actions required to reach desirable results [28]. The model gives an insight into how self-efficacy beliefs about physical activity during cancer treatment can influence behavior. Beliefs of self-efficacy are based on actual/mastery experience, vicarious/observational experience, verbal persuasion and emotional arousal (physiological states and affective states). In the PACT supervised exercise program, we addressed the first three determinants. For actual/mastery experience, patients were asked to report their training results in an exercise log. Vicarious/observational experiences included modelling (the use of a role model) and a DVD showing the exercise experiences of other cancer patients. Verbal persuasion included a leaflet showing the effectiveness of exercise in other patients and the supervision by the physiotherapist during the sessions. Physiological states and affective states are part of emotional arousal. Emotional arousal was not addressed in the exercise program, because it can be better targeted through other interventions like cognitive behavioral therapy.

Patients randomized into the control group (not included in the present analysis) received the usual care and were asked to maintain their habitual physical activity patterns up to week 18.

### Outcome measures

**Fig 2:**
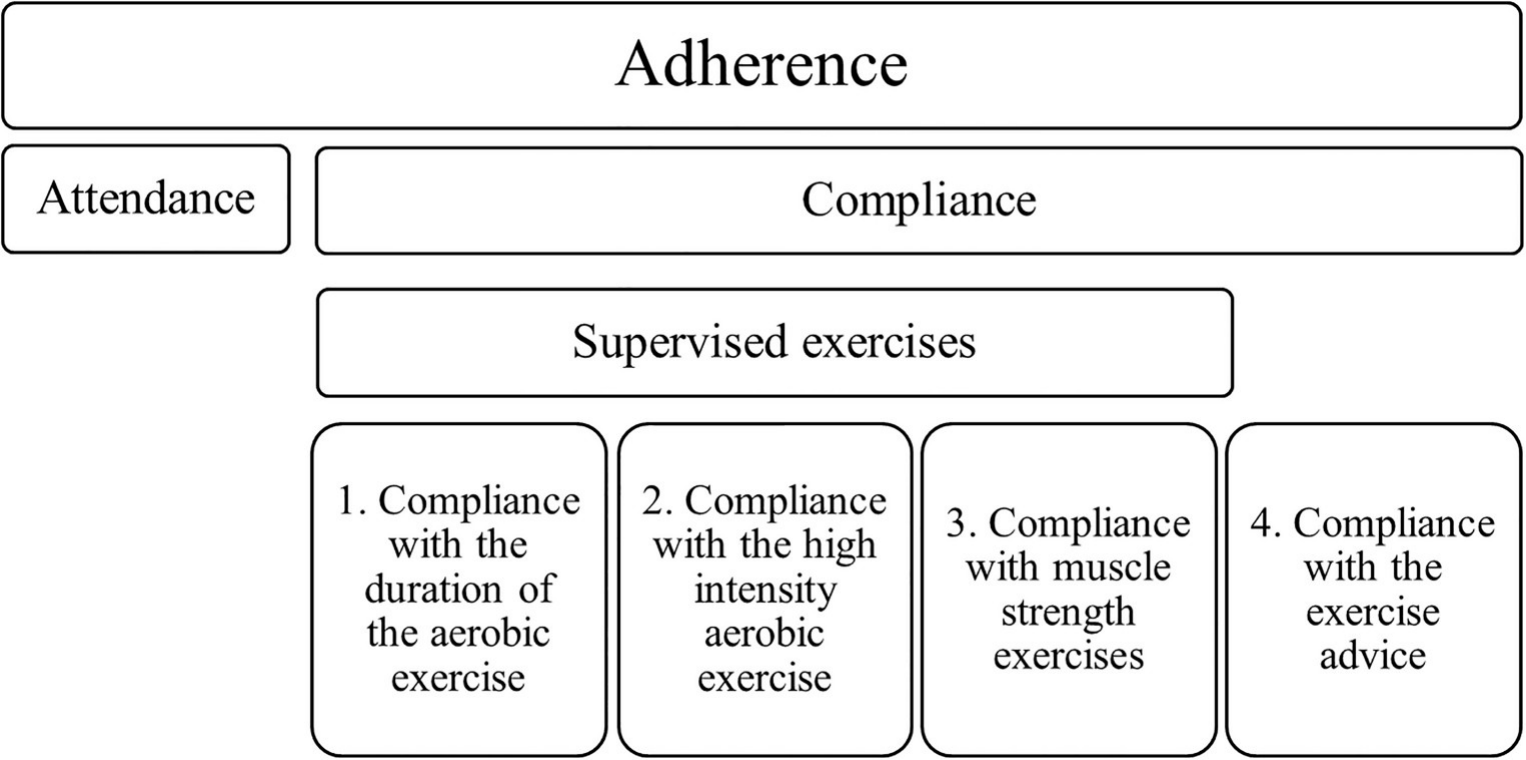
Overview of the outcome measures for adherence.

The primary outcome in the present study was adherence, which incorporates both attendance and compliance rates with the PACT intervention. Attendance is simply scoring if someone is present or not independent what the patient during the exercise session. The next step is to assess whether the session was performed according to the exercise protocol, which we defined as compliance. Attendance rates were computed for each patient as the number of supervised sessions attended divided by the number of sessions offered. Compliance rates were computed individually for four measures of the PACT program: (1) the duration of aerobic exercises, (2) the intensity of aerobic exercises, (3) the muscle strength exercises, and (4) compliance with the exercise advice (Fig 2).

#### Attendance

Patient attendance of the 36 supervised sessions in the PACT intervention was registered in a Case Record Form by the physiotherapist.

#### Compliance with the supervised exercise program

During each exercise session, patients registered their performed exercises in an exercise log supervised by the physiotherapist. They reported the total duration of the aerobic exercises in minutes, as well as the duration of the high-intensity intervals (i.e., the number of minutes at or above the VT). The weight used and number of repetitions performed for all muscle strength exercises were also registered. Compliance with the following elements were determined per patient in percentages:

- The duration of the aerobic exercises, i.e. performing the total prescribed number of minutes;
- The intensity of the aerobic exercises, i.e. performing the prescribed number of minutes at or above the VT;
- The muscle strength exercises, i.e. at least three of the five prescribed exercises according to the recommended intensity (weight multiplied by the number of repetitions). We set three as the maximum since an adaption of the protocol was allowed for two exercises because not all locations were suitable for the performance of 1-RM tests for all exercises, due to the limitations of the equipment.

#### Compliance with the exercise advice

Patients registered the duration of activities they performed at a moderate-to-high intensity in their activity diary on a daily basis. We defined moderate-to high intensity activities according to the Dutch guidelines for physical activity, including activities with a MET-value of at least 4.0 (walking and hiking were also included because at baseline patients were explicitly instructed to register only when walking at a steady pace and when walking at a steady pace and when they had difficulties talking at the same time) [31, 32]. The number of weeks in which patients met the advice for five days of exercise were determined using these diaries [27].

### Assessment of predictors

Our second aim was to assess predictors of adherence. The following predictors, which were assessed at baseline, were considered: demographical factors, tumor and treatment factors, constructs of the theory of planned behavior, psychological factors and physical factors.

#### Demographical factors

Self-reported data were collected for the following factors: age (years), education (high: higher vocational and university education; medium: secondary and secondary vocational education; low: elementary and lower vocational education), and marital status (living alone versus living together).

#### Tumor and treatment factors

Radiotherapy treatment status (yes or no) and tumor receptor status (triple negative, Her2Neu+ with ER/PR +/-, or Her2Neu- with ER/PR+) were retrieved from medical records. Tumor receptor status was a proxy for treatment type and treatment duration, since actual treatment plan would result in a variable with too many categories.

#### Constructs of the theory of planned behavior

Ajzen’s theory of planned behavior is the most commonly used framework to understand exercise behavior [24, 33, 34]. The theory links beliefs and behavior. The patients’ beliefs about attending 30 out of 36 exercise sessions and beliefs about being physically active according to the guidelines were assessed as specified by the theory of planned behavior: intention, subjective norm, perceived behavioral control and attitude [24]. The subscales were recorded on a 10-point Likert scale.

#### Psychological factors

Anxiety and depression were assessed using the validated Dutch language version of the Hospital Anxiety and Depression Scale (HADS) [35]. This questionnaire consists of 14 items; seven items on the depression subscale and seven items on the anxiety subscale resulting in a total depression score and a total anxiety score (both scored 0 (no depressive symptoms/anxiety) to 21 (depressive symptoms/anxiety)).

Health-related quality of life was assessed using the global health status subscale of the European Organisation for Research and Treatment of Cancer Quality of Life Questionnaire C30 (EORTC QLQ-C30) [36]. The global health status subscale consists of two items, regarding overall health and overall quality of life, and ranges from 0 (low score global health status) to 100 (high score global health status).

Self-efficacy was assessed using seven (attending 30 sessions or more) or eight (being physically active for at least 30 minutes for five days per week) items, based upon the social cognitive theory of Bandura [28]. Self-efficacy items were assessed on a 10-point Likert scale with endpoints labelled ‘strongly disagree’ and ‘strongly agree’. Self-efficacy scores range form 1–10, where a higher score means a high belief about self-efficacy for the behavior.

#### Physical factors

Body weight and height were measured to the nearest 0.5 kg and 0.5 cm, respectively, with the subjects wearing light clothes and no shoes. These data were used to calculate body mass index (BMI; kg/m^2^).

Physical Activity was measured using the Short Questionnaire to Assess Health-Enhancing Physical Activity (SQUASH). Baseline levels of moderate-to-high intensity leisure and sport activities were calculated in minutes per week for a typical week before diagnosis [37].

Physical fatigue was measured using a subscale of the validated Multidimensional Fatigue Inventory (MFI) [38]. The MFI is a self-report instrument designed to measure multiple fatigue characteristics and the impact on function. The score on the physical fatigue subscale ranges from 4 to 20, with a higher score indicating greater fatigue.

Cardiorespiratory fitness (peak oxygen consumption; liters per minute) was determined using a CPET for patients experiencing an increasing workload on a cycle ergometer with continuous gas analysis of breath. The test was terminated in response to the patients’ symptoms or at the physician’s discretion. The mean of the VO_2_ values of the last 30 seconds before exhaustion was determined as peak oxygen consumption.

### Statistical analyses

Descriptive statistics were used to describe the patients’ characteristics and baseline values of potential predictors. The attendance and compliance rates are reported as medians with interquartile ranges. Descriptive statistics were shown for the total group, and were also stratified for patients attending 0–19 (0–53%), 20–29 (54–80%) and 30–36 (81–100%) sessions in order to explore whether patients with higher attendance rates differed from patients attending fewer exercise sessions.

A univariable linear regression was used to predict the rate of attendance and compliance in order to identify possible predictors for multivariable model building using an intention to treat analysis. The selection of possible predictors was based on p<0.20, and a Pearson’s correlation coefficient of <0.7 among each other. The possible predictors were included in the multivariable model, and the final model was obtained by performing a multivariable linear regression with the stepwise backward selection of factors based on Akaike’s information criterion at p<0.157. Similar p-values are commonly used in prediction research [39]. Predictors are reported as estimated unstandardized regression coefficients (β) with a 95% confidence interval (CI). Model fit of the multivariable model was expressed as R^2^. All analyses were performed using SPSS version 21.0.

As stated before, the results should be interpreted as exploratory data. Because of this, adjusting for multiple comparisons was not done.

## Results

### Baseline characteristics

The 92 patients had a mean age of 50.2 ± 7.8 (standard deviation) years and an average BMI of 25.7 ± 4.4 kg/m^2^. Sixty-four patients (69.6%) received radiotherapy in addition to chemotherapy (Table 1). Patients reported to be physically active for more than 30 minutes a day on 4.9 ± 2.1 days per week prior to diagnosis. Patients who attended fewer than 20 of the prescribed exercise sessions had, on average, a higher BMI, lower quality of life, lower educational level, received more radiotherapy and were more physically fatigued compared with patients who attended more sessions.

**Table 1:** Baseline characteristics of 92 breast cancer patients, reported for the total group and stratified according to attendance.

Predictor	Total Group (n = 92)	0–19 sessions attended (n = 12)	20–29 sessions attended (n = 33)	30–36 sessions attended (n = 47)	Exercise advise group (n = 82)
**Demographical**					
Age (years (mean ± SD))	50.2 ± 7.8	51.5 ± 5.1	47.8 ± 9.2	51.6 ± 6.9	50.0 ± 7.9
Educational status (n (%))					
Low	4 (4.3%)	3 (25.0%)	0 (0.0%)	1 (2.1%)	2(2.4%)
Medium	44 (47.8%)	6 (50.0%)	17 (51.5%)	21 (44.7%)	40 (48.8%)
High	42 (45.7%)	1 (8.3%)	16 (48.5%)	25 (53.2%)	40 (48.8%)
*Missing*	*2 (2*.*2%)*	*2 (16*.*7%)*	*0 (0*.*0%)*	*0 (0*.*0%)*	*0 (0*.*0%)*
Marital status (n (%))					
Together	73 (79.3%)	8 (66.7%)	26 (78.8%)	39 (83.0%)	66 (80.5%)
Alone	17 (18.5%)	2 (16.7%)	7 (21.2%)	8 (17.0%)	16 (19.5%)
*Missing*	*2 (2*.*2%)*	*2 (16*.*7%)*	*0 (0*.*0%)*	*0 (0*.*0%)*	*0 (0*.*0%)*
**Tumor and treatment**					
Radiotherapy (n (%))	64 (69.6%)	10 (83.3%)	23 (69.7%)	31 (66.0%)	55 (67.1%)
Receptor status (n (%))					
Triple negative	23 (25.0%)	2 (16.7%)	10 (30.3%)	11 (23.4%)	21 (25.6%)
Her2+ and ER+ or PR+	8 (8.7%)	0 (0.0%)	4 (12.1%)	4 (8.5%)	8 (9.8%)
Her2+ and ER- and PR-	10 (10.9%)	1 (8.3%)	3 (9.1%)	6 (12.8%)	10 (12.2%)
Her2- and ER+ or PR+	51 (55.4%)	9 (75.0%)	16 (48.5%)	26 (55.3%)	43 (52.4%)
	**Mean ± SD**	**Mean ± SD**	**Mean ± SD**	**Mean ± SD**	**Mean ± SD**
**Theory of planned behavior**					
Beliefs about attending ≥ 30 exercise sessions					
Intention	9.3 ± 1.2	8.8 ± 1.3	9.3 ± 1.2	9.4 ± 1.1	9.4 ± 1.1
Subjective norm	5.9 ± 1.9	5.5 ± 2.5	5.9 ± 1.7	6.0 ± 1.9	5.9 ± 1.9
Perceived behavioral control	8.0 ± 1.5	7.8 ± 1.4	7.8 ± 1.5	8.1 ± 1.5	7.9 ± 1.5
Attitude	8.7 ± 1.5	8.4 ± 1.8	9.0 ± 1.3	8.6 ± 1.5	8.7 ± 1.4
Beliefs about being physically active for ≥ five days per week					
Intention	8.8 ± 1.3	8.4 ± 1.8	8.6 ± 1.4	9.1 ± 1.0	8.8 ± 1.3
Subjective norm	5.4 ± 1.8	5.4 ± 2.1	5.5 ± 1.8	5.3 ± 1.7	5.4 ± 1.7
Perceived behavioral control	7.9 ± 1.5	7.9 ± 1.6	7.7 ± 1.8	8.0 ± 1.4	7.8 ± 1.6
Attitude	8.5 ± 1.7	8.3 ± 1.8	8.6 ± 1.7	8.4 ± 1.7	8.4 ± 1.7
**Psychological**					
Anxiety	4.5 ± 3.4	4.6 ± 3.8	5.2 ± 4.1	4.0 ± 2.8	4.4 ± 3.4
Depression	2.6 ± 3.2	2.9 ± 3.6	3.0 ± 3.3	2.2 ± 2.9	2.7 ± 3.1
Health-related quality of life	74.5 ± 21.1	66.7 ± 30.4	75.5 ± 19.6	75.9 ± 19.3	75.5 ± 19.4
Beliefs about self-efficacy					
Attending ≥ 30 sessions	7.1 ± 1.9	7.3 ± 1.5	7.2 ± 2.0	7.0 ± 1.9	7.0 ± 1.8
Physical activity ≥ five days per week	7.0 ± 2.0	7.3 ± 1.9	6.7 ± 2.3	7.2 ± 1.9	6.9 ± 2.0
**Physical**					
BMI (kg/m^2^)	25.7 ± 4.4	29.0 ± 4.9	26.0 ± 4.9	24.7 ± 3.5	25.4 ± 4.2
Baseline physical activity (minutes/week)	482,89 ± 449.9	163.3 ± 184.7	199.2 ± 257.2	376.1 ± 573.3	284.4 ± 466.6
Physical fatigue	9.9 ± 4.4	12.1 ± 5.1	10.2 ± 4.5	9.2 ± 4.0	10.0 ± 4.4
Peak oxygen consumption (L/min)	1.7 ± 0.3	1.6 ± 0.3	1.7 ± 0.4	1.7 ± 0.3	1.7 ± 0.3

### Attendance

The median attendance rate for patients was 83% (interquartile range: 69% to 91%); 13% attended fewer than 20 of the supervised exercise sessions, 36% attended 20 to 29 sessions, and 51% attended 30 or more.

### Compliance

Compliance with the three measures of the supervised program were 88% (63% to 97%) for the duration of aerobic exercise, 50% (22% to 82%) for the high-intensity aerobic exercises, and 84% (65% to 94%) for the muscle strength exercises (Table 2). Three patients complied with all three components of the supervised program for 30 sessions or more. Patients who attended 19 sessions or fewer had a lower compliance with all three components of the supervised exercise program than those who attended 30 or more sessions.

**Table 2:** Compliance rates according to the four domains of the PACT intervention.

	Total group (n = 92)		0–19 exercise sessions attended (n = 12)		20–29 exercise sessions attended (n = 33)		30–36 exercise sessions attended (n = 47)	
Compliance* with the…	Median	IQR	Median	IQR	Median	IQR	Median	IQR
Supervised exercises								
Duration of aerobic exercise^$^	87.5%	63.3–96.8%	59.7%	35.4–88.1%	87.5%	65.5–96.4%	90.9%	77.4–97.0%
High-intensity aerobic exercise^$^	49.8%	22.1–81.6%	36.6%	17.8–68.0%	41.4%	21.4–77.4%	63.3%	29.4–86.1%
Muscle strength exercise^$^	83.9%	65.2–93.7%	63.3%	28.4–82.2%	87.5%	69.2–96.1%	83.9%	73.3–93.8%
Exercise advice ^#^	61.1%	33.3–79.1%	33.3%	8.3–58.3%	47.2%	22.2–70.8%	66.7%	55.6–88.9%

* Compliance was defined as following the prescribed exercise protocol

^$^ Numbers were computed as the (number of sessions complied with)/(number of sessions attended)

^#^ Numbers were computed as the (number of weeks complied with)/(number of weeks of exercise program)

Compliance with the exercise advice was 61% (33% to 79%) and seven patients (9%) fully complied (i.e., completed more than 30 minutes of moderate-to-high intensity physical activity on at least five days a week for 18 consecutive weeks). Compliance with the exercise advice was lower in patients who attended fewer than 20 supervised exercise sessions (33% (8% to 58%)) compared with those attending 30 or more sessions (67% (56% to 89%)) (Table 2).

### Predictors of attendance

All univariable results are reported in S2 Table.

The multivariable model retrieved four predictors for attendance: higher educational level predicted a higher attendance (β = 8.24 (95% CI: 0.93, 15.54)); however, receiving radiotherapy in addition to chemotherapy (β = -7.08 (95% CI: -14.91, 0.74), having a high BMI (β = -1.36 (95% CI: -2.21, -0.52)) and high physical fatigue level (β = -0.61 (95% CI: -1.45, 0.24)) predicted low attendance.

### Predictors of compliance

In the multivariable analysis, a higher peak oxygen consumption (β = 5.67 (95% CI: -0.27, 11.61)) predicted a higher compliance with the duration of the aerobic exercises, whereas patients with higher self-efficacy beliefs about attending 30 sessions or more (β = -0.89 (95% CI: -1.99, 0.21)), a higher BMI (β = -0.44 (95% CI: -0.89, 0.01)) and higher physical fatigue (β = -0.34 (95% CI: -0.81, 0.12)) showed lower compliance with the duration of aerobic exercises (Table 3).

**Table 3:** Multivariable linear regression analyses on the association between selected predictors and attendance and compliance.

	Attendance		Compliance with duration of aerobic exercise		Compliance with high-intensity aerobic exercise		Compliance with muscle strength exercise		Compliance with exercise advice	
	Total group (n = 92)		Total group (n = 92)		Total group (n = 92)		Total group (n = 92)		Total group (n = 82)	
Predictor	β (95% CI)	P	β (95% CI)	P	β (95% CI)	P	β (95% CI)	P	β (95% CI)	P
**Demographical**										
Age (years)	#	#	-	-	-0.22 (-0.47, 0.02)	0.08	#	#	#	#
Educational status										
high vs. low and medium	8.24 (0.93, 15.54)	0.03	-	-	2.93 (-0.85, 6.70)	0.13	-	-	#	#
**Tumor and treatment**										
Radiotherapy	-7.08 (-14.91, 0.74)	0.08	#	#	-5.32 (-9.36, -1.28)	0.01	#	#	#	#
Tumor receptor status										
Her2+ and ER or PR+ vs. Triple negative	#	#	#	#	#	#	6.63 (-0.44, 13.70)	0.07	#	#
Her2- and ER or PR+ vs. Triple negative	#	#	#	#	#	#	#	#	1.55 (-0.47; 3.57)	0.13
**Theory of planned behavior**										
**Beliefs about physical activity for ≥5 days per week**										
Intention	NA	NA	NA	NA	NA	NA	NA	NA	0.80 (0.00, 1.60)	0.05
Subjective norm	NA	NA	NA	NA	NA	NA	NA	NA	0.59 (0.00, 1.18)	0.05
Perceived behavioral control	NA	NA	NA	NA	NA	NA	NA	NA	-	-
**Psychological**										
Anxiety	#	#	-	-	#	#	-	-	#	#
Depression	#	#	-	-	#	#	-	-	#	#
Health-related quality of life	-	-	-	-	-	-	-	-	#	#
Beliefs about self-efficacy										
Attending 30 sessions	#	#	-0.89 (-1.99, 0.21)	0.11	#	#	#	#	-	-
**Physical**										
BMI (kg/m^2^)	-1.36 (-2.21, -0.52)	0.002	-0.44 (-0.89, 0.01)	0.06	-	-	-0.43 (-0.88, 0.03)	0.07	#	#
Baseline physical activity	-	-	#	#	0.00 (0.00;0.01)	0.11	0.00 (0.00,0.01)	0.09	0.00 (0.00;0.01)	0.05
Baseline physical fatigue	-0.61 (-1.45, 0.24)	0.156	-0.34 (-0.81, 0.12)	0.15	-0.47 (0.90;-0.04)	0.03	-	-	#	#
Peak O_2_ consumption (L/min)	#	#	5.67 (-0.27, 11.61)	0.06	-	-	-	-	3.65 (0.49, 6.80)	0.02
**R square**	0.20		0.16		0.20		0.09		0.24	

Estimated unstandardized regression coefficients with 95% Confidence intervals are mentioned in this table. # Not significant in univariate analysis.—Significant in univariate analysis, however not significant in multivariable analysis. NA: Not applicable

For compliance with the prescribed high intensity part of the aerobic exercises, a higher educational level (β = 2.93 (95% CI: -0.85, 6.70)) and higher baseline physical activity level (β = 0.00(95%CI: 0.00, 0.01)) resulted in higher compliance. Older patients (β = -0.22 (95% CI: 0.47, 0.02), those reporting higher levels of physical fatigue (β = -0.47 (95% CI: -0.90, -0.04)) as well as patients who received radiotherapy in addition to chemotherapy (β = -5.32 (95% CI: -9.36, -1.28)), had a lower compliance.

Higher compliance with the muscle strength exercises was predicted by having a Her2+ and ER or PR+ tumor receptor status versus a triple negative receptor status (β = 6.63 (95% CI: -0.44, 13.70)), a lower BMI (β = -0.43 (95% CI: -0.88, 0.03)) and baseline physical activity level (β = 0.00 (95% CI: 0.00,0.01).

Having a Her2- and ER or PR+ tumor receptor status versus a triple negative receptor status (β = 1.55 (95% CI: -0.47, 3.57), a higher peak oxygen consumption (β = 3.65 (95% CI: 0.49, 6.80)), a higher intention (β = 0.80 (95% CI: 0.00, 1.60)), higher baseline physical activity level (β = 0.00 (95% CI: 0.00, 0.01)) and a higher subjective norm to be physically active for at least five days per week (β = 0.59 (95% CI: 0.00, 1.18)) predicted a higher compliance with the exercise advice.

## Discussion

This explorative study shows high adherence rates to an 18-week aerobic and muscle strength exercise program in primary breast cancer patients during treatment, both in terms of attendance rates and compliance rates, with the exception of compliance to the high intensity exercises. The attendance rate (83%) was higher than in previous trials investigating the use of exercise programs in breast cancer patients undergoing chemotherapy, whose attendance rates of twice weekly supervised sessions ranged from 71–79% [15–17]. This might be explained by the incorporation of cognitive behavioral aspects into our exercise program or differences in patient characteristics, such as baseline activity levels, which were high in the PACT population. In addition, the intensity of supervision might have differed. In the PACT study, patients either trained in small groups or alone, and the physiotherapists explicitly encouraged the patients to attend the sessions even though they were not feeling well. Note, that physiotherapists are exercise specialists and internationally other allied health trained exercise professionals can adopt and use this intervention.

If adjustments were necessary in this study, they were made in the high-intensity period of the aerobic supervised exercises, resulting in lower compliance. In this study, the intensity interval training consisted for 2 intervals of 2 to 7 minutes. This high intensity training is longer than other high intensity interval training protocols used in similar trials [40]. It might be that patients would experience the shorter work-rest ratio’s more tolerable and enjoyable which might explain the lower compliance with the high intensity period of the aerobic supervised exercises in our study.

The goal of exercise program was to follow the protocol as precisely as possible. If adjustments were necessary because of wellbeing of the patients, we considered this as a lower compliance. We believe that this is sometimes medically or ethically appropriate, since the patients underwent intensive treatment with chemotherapy. However, we wanted to know if this original protocol was doable for the patients. We would have found higher compliance rates if we had counted adjustments because of medical or ethical reasons as ‘compliant’. In future studies, it might be interesting to investigate whether autoregulation, i.e. allow patients to do less when they feel unwell and do more if they feel well, improves compliance rates.

### Factors contributing to higher adherence

We found in this additional explanatory analysis several predictors for both attendance and compliance in breast cancer patients. In this study, a higher educational level, not receiving radiotherapy, a lower BMI and less physical fatigue predicted higher attendance. Other studies in breast cancer patients undergoing treatment found various other predictors of adherence to different exercise prescriptions; a higher peak oxygen uptake, fewer endocrine symptoms, lower durations of exercise, fewer exercise limitations, shorter chemotherapy protocols and exercise facility location all contributed to higher levels of attendance [12, 13]. The authors concluded that predictors of attendance in breast cancer patients are diverse and may differ as a result of the particular exercise prescriptions [12, 13], which also explains the differences between these findings and those of the present study. In addition, the set of possible predictors were not identical across all studies.

To the best of our knowledge, there have been no reports on the predictors of compliance with a supervised exercise program during breast cancer treatment. In our study, physical fatigue predicted a lower compliance with the duration of aerobic exercise and with the high-intensity periods of aerobic exercise, but not with the muscle strength exercises. It might be possible that the physiotherapists first adjusted the aerobic exercises, implying the patients were probably still able to perform the muscle strength exercises despite being fatigued. We did not ask specific reasons for the choice of adjusting the intensity, but our data from the exercise logs suggest that, in general, adjustments were made mainly due to the side effects of treatment such as nausea, fatigue and dizziness.

Patients had a higher compliance with the duration of the aerobic exercises when the peak oxygen consumption was higher. These patients had probably a more physically active lifestyle and were therefore more used to exercise and able to continue the aerobic exercises.

In this study, a remarkable finding was that compliance with the duration of aerobic exercises was lower in patients who reported high self-efficacy beliefs about attending 30 sessions or more at the baseline. It might be that these patients overestimated their own capabilities about physical exercise during cancer treatments; however, this did not affect their compliance with any of the other aspects of the exercise program. On the other hand, our question did not address the patients’ belief of whether they will be able to follow the specific exercises but about their self-efficacy beliefs about attending the sessions.

Patients receiving radiotherapy were less compliant with the high-intensity aerobic exercise. One speculative explanation might be the burden of daily traveling to the hospital for the radiotherapy in combination with attending the PACT exercise sessions, which were not generally held at the same location.

Huang et al. investigated predictors for compliance with both the intensity and duration of a home-based walking program in breast cancer patients [18]. Patients who were less fatigued, perceived a higher importance of exercise, had an early stage of disease and were employed had a higher compliance with exercise intensity. Our study showed that patients who were more physically fit and had a higher intention and higher subjective norm towards being physically active for 30 minutes at least five days per week were more compliant with the exercise advice (i.e., the home-based aspects of the program). In this study, it seems that motivational factors play a more important role in the home-based exercise program than for the supervised exercise program, however this is unclear in previous research [19]. The patient’s beliefs about being physically active in a home-based setting during cancer treatment might be more influenced by the social support within the patient’s environment, such as from a partner, compared with the supervised exercise setting. On the other hand, marital status was not found as a predictor for compliance with the exercise advice. Since we only included marital status in our analysis and no other possible social support from family, friends or carers, this should be included in future trials. Future studies should include more extended information on social support. During supervised exercise, the physiotherapist or other allied health trained exercise professionals and other patients may influence the patient’s motivation; therefore, the patient’s own motivational factors and social context may have been less important [41].

### Strengths and limitations

The strengths of the present study are the detailed registration of the different components of compliance and the availability of information on several predictors, including demographical, tumor-, and treatment-related factors, constructs of the theory of planned behavior, psychological and physical factors. The PACT study was a pragmatic trial that resembles daily practice, which increases the generalizability of results. On the other hand, although we cannot exclude overreporting in the questionnaires, patients who participated in this study reported rather high average physical activity levels at the baseline. This probably influenced the generalizability of the predictors to the total population. Furthermore, information about compliance with the exercise advice was extracted from self-reported activity diaries rather than using objective measures and only 80% of the original 102 patients returned their diaries. An overestimation of the performed activities might be reported in the activity diaries, although we explicitly instructed the patients at baseline to fill in activities such as walking or cycling only when they had difficulties talking at the same time. For the supervised sessions, it was not registered whether the high-intensity intervals were adjusted at request of the patient or the physiotherapist. Our analyses should be interpreted as exploratory, since the study was not primarily powered for the current study; consequently, additional predictors might have been missed. In other studies, exercise facility location [20], fewer endocrine symptoms [13], lower durations of exercise [13], fewer exercise limitations [13], shorter chemotherapy protocol [13], exercise history [19], being employed[18, 21], high income [21], early stage disease [18] and being Caucasian [22] were found as predictors for adherence, while these predictors were not assessed in this study. In univariate analyses, several predictors were found to be significant but were not supported in the multivariable analysis, which might have been the result of multicollinearity or restricted statistical power. These unmeasured predictors might also influence attendance and compliance.

### Conclusions and clinical implications

The high attendance and compliance rates reported in this study suggest that both supervised aerobic and resistance exercise and exercise advice are feasible for localized breast cancer patients undergoing adjuvant treatment. We observed that high-intensity aerobic exercises were adjusted for a significant number of patients, in preference to making changes to the duration or the strength exercises. Particularly for older patients, patients who were more fatigued at the baseline, patients having a lower educational level or receiving radiotherapy led to difficulties in complying with the high-intensity aerobic exercises. Future studies should consider shorter intervals of high intensity aerobic exercises which might increase compliance rates. Beliefs about exercise behaviors are important for compliance with the exercise advice. The predictors we have identified should be taken into account when designing future exercise programs or adapting present programs for breast cancer patients, to increase compliance. Choosing training locations in the immediate vicinity of the patient’s home and the availability of social support from, for example, a partner or general practitioner, might be additional factors that could increase adherence.

## Supporting information

S1 Table Protocol for the supervised exercise sessions. *High intensity: at or above ventilator threshold. **Low intensity: under ventilatory threshold.
(DOCX)

S2 Table Univariate linear regression analyses on the association between the predictors and attendance and compliance. Bold is significant (p<0.2); Estimated unstandardized regression coefficients with 95% Confidence intervals are mentioned in this table. NA: not applicable.
(DOCX)

S1 File Onderzoeksprotocol Op weg naar herstel_amendement 261110. (PDF)

S2 File CONSORT PLOS one. (DOC)
