# Supplementary material for: Attendance and compliance with an exercise program during localized breast cancer treatment in a randomized controlled trial: The PACT study
Source: PLoS One. 2019 May 8;14(5):e0215517. doi: 10.1371/journal.pone.0215517 (PMC6505930; doi:10.1371/journal.pone.0215517)
Supplement: S1 Table — *High intensity: at or above ventilator threshold. **Low intensity: under ventilatory threshold. (DOCX) [file pone.0215517.s001.docx]

|  |  | **Total aerobic exercise** | | | **Muscle strength exercise** | |
| --- | --- | --- | --- | --- | --- | --- |
|  |  | **Time at intensity** | | **Total duration** |  |  |
| **Week** | **Session number** | **Time at high intensity*** | **Time at low intensity**** | **Time at low  and high intensity** | **Repetitions** | **Percentage of 1-RM** |
| 1 | 1 | 3 x 2 minutes | 3 x 4 minutes | 18 minutes | **1-RM test** | |
|  | 2 | 3 x 2 minutes | 3 x 4 minutes | 18 minutes | 2 x 10 | 65% |
| 2 | 3 | 2 x 3 minutes | 2 x 6 minutes | 18 minutes | 2 x 8-12 | 65% |
|  | 4 | 2 x 3 minutes | 2 x 6 minutes | 18 minutes | 2 x 8-12 | 65% |
| 3 | 5 | 2 x 4 minutes | 2 x 6 minutes | 20 minutes | 2 x 8-12 | 70% |
|  | 6 | 2 x 4 minutes | 2 x 6 minutes | 20 minutes | 2 x 8-12 | 70% |
| 4 | 7 | 2 x 5 minutes | 2 x 5 minutes | 20 minutes | 2 x 8-12 | 75% |
|  | 8 | 2 x 5 minutes | 2 x 5 minutes | 20 minutes | 2 x 8-12 | 75% |
| 5 | 9 | **1-RM test / Aerobic evaluation test** | | | | |
|  | 10 | 2 x 6 minutes | 1 x 8 minutes | 20 minutes | 2 x 8-12 | 70% |
| 6 | 11 | 2 x 6 minutes | 1 x 8 minutes | 20 minutes | 2 x 8-12 | 70% |
|  | 12 | 2 x 6 minutes | 1 x 8 minutes | 20 minutes | 2 x 8-12 | 70% |
| 7 | 13 | 2 x 7 minutes | 1 x 7 minutes | 21 minutes | 2 x 8-12 | 75% |
|  | 14 | 2 x 7 minutes | 1 x 7 minutes | 21 minutes | 2 x 8-12 | 75% |
| 8 | 15 | 2 x 7 minutes | 1 x 7 minutes | 21 minutes | 2 x 8-12 | 80% |
|  | 16 | 2 x 7 minutes | 1 x 7 minutes | 21 minutes | 2 x 8-12 | 80% |
| 9 | 17 | **1-RM test / Aerobic evaluation test** | | | | |
|  | 18 | 2 x 7 minutes | 1 x 7 minutes | 21 minutes | 2x 20 | 35% |
| 10 | 19 | 2 x 7 minutes | 1 x 7 minutes | 21 minutes | 2 x 8-12 | 70% |
|  | 20 | 2 x 7 minutes | 1 x 7 minutes | 21 minutes | 2 x 16-24 | 40% |
| 11 | 21 | 2 x 7 minutes | 1 x 7 minutes | 21 minutes | 2 x 8-12 | 75% |
|  | 22 | 2 x 7 minutes | 1 x 7 minutes | 21 minutes | 2 x 16-24 | 45% |
| 12 | 23 | 2 x 7 minutes | 1 x 7 minutes | 21 minutes | 2 x 8-12 | 75% |
|  | 24 | 2 x 7 minutes | 1 x 7 minutes | 21 minutes | 2 x 16-24 | 45% |
| 13 | 25 | 2 x 7 minutes | 1 x 7 minutes | 21 minutes | 2 x 8-12 | 75% |
|  | 26 | 2 x 7 minutes | 1 x 7 minutes | 21 minutes | 2 x 16-24 | 45% |
| 14 | 27 | **1-RM test / Aerobic evaluation test** | | | | |
|  | 28 | 2 x 7 minutes | 1 x 7 minutes | 21 minutes | 1 x 8-12 & 1 x 16-24 | Resp. 65% & 35% |
| 15 | 29 | 2 x 7 minutes | 1 x 7 minutes | 21 minutes | 1 x 8-12 & 1 x 16-24 | Resp. 65% & 35% |
|  | 30 | 2 x 7 minutes | 1 x 7 minutes | 21 minutes | 1 x 8-12 & 1 x 16-24 | Resp. 65% & 35% |
| 16 | 31 | 2 x 7 minutes | 1 x 7 minutes | 21 minutes | 1 x 8-12 & 1 x 16-24 | Resp. 70% & 40% |
|  | 32 | 2 x 7 minutes | 1 x 7 minutes | 21 minutes | 1 x 8-12 & 1 x 16-24 | Resp. 70% & 40% |
| 17 | 33 | 2 x 7 minutes | 1 x 7 minutes | 21 minutes | 1 x 8-12 & 1 x 16-24 | Resp. 75% & 45% |
|  | 34 | 2 x 7 minutes | 1 x 7 minutes | 21 minutes | 1 x 8-12 & 1 x 16-24 | Resp. 75% & 45% |
| 18 | 35 | 2 x 7 minutes | 1 x 7 minutes | 21 minutes | 1 x 8-12 & 1 x 16-24 | Resp. 75% & 45% |
|  | 36 | 2 x 7 minutes | 1 x 7 minutes | 21 minutes | 1 x 8-12 & 1 x 16-24 | Resp. 75% & 45% |

**S1 Table:** **Protocol for the supervised exercise sessions**

*High intensity: at or above ventilatory threshold. **Low intensity: under ventilatory threshold
