# Supplementary material for: Attendance and compliance with an exercise program during localized breast cancer treatment in a randomized controlled trial: The PACT study
Source: PLoS One. 2019 May 8;14(5):e0215517. doi: 10.1371/journal.pone.0215517 (PMC6505930; doi:10.1371/journal.pone.0215517)
Supplement: S2 Table — Bold is significant (p<0.2); Estimated unstandardized regression coefficients with 95% Confidence intervals are mentioned in this table. NA: not applicable. (DOCX) [file pone.0215517.s002.docx]

|  | | **Attendance** |  | **Compliance with the duration of aerobic exercise** | | **Compliance with the high intensity aerobic exercise** | | **Compliance with the muscle strength exercise** | | **Compliance with the exercise advice** | |
| --- | --- | --- | --- | --- | --- | --- | --- | --- | --- | --- | --- |
|  | | Total group (n=92) |  | Total group (n=92) | | Total group (n=92) | | Total group (n=92) | | Total group (n=82) | |
| Predictor | | β (CI-95) | P | β (CI-95) | P | β (CI-95) | P | β (CI-95) | P | β (CI-95) | P |
| Demographical | |  |  |  |  |  |  |  |  |  |  |
| Age (years) | | -0.10 (-0.65; 0.46) | 0.726 | **-0.17 (-0.43;0.09)** | **0.189** | **-0.23 (-0.49;0.03)** | **0.079** | 0.13 (-0.13;0.39) | 0.313 | 0.07 (-0.07;0.22) | 0.307 |
| Educational status | |  |  |  |  |  |  |  |  |  |  |
|  | high vs. low & medium | **-9.66 (1.86; 17.47)** | **0.016** | **3.04 (-0.87;6.96)** | **0.126** | **3.25 (-0.74;7.25)** | **0.109** | 1.84 (-2.12;5.79) | 0.358 | -0.54 (-2.78;1.70) | 0.632 |
| Marital status, alone vs. together | | -3.00 (-13.26; 7.27) | 0.563 | -1.47 (-6.52;3.58) | 0.565 | -1.05 (-6.20;4.11) | 0.688 | -0.42 (-5.48;4.64) | 0.870 | 0.22 (-2.61;3.04) | 0.879 |
| Tumor and treatment | |  |  |  |  |  |  |  |  |  |  |
| Radiotherapy | | **-6.10 (-15.30; 3.09)** | **0.191** | -2.66 (-6.99;1.68) | 0.227 | **-4.88 (-9.13;-0.64)** | **0.025** | -2.54 (-6.87;1.80) | 0.248 | 0.73 (-1.65;3.10) | 0.544 |
| Tumor receptor status | |  |  |  |  |  |  |  |  |  |  |
|  | Her2+ & ER or PR+ vs. Triple - | 0.61 (-15.77;14.55) | 0.937 | 3.67 (-3.43;10.77) | 0.308 | 3.25 (-3,85;10.35) | 0.365 | **4.89 (-2.17;11.95)** | **0.172** | -0.36 (-4.13;3.41) | 0.849 |
|  | Her2+ & ER & PR- vs. Triple - | 4.55 (-9.14;18.24) | 0.511 | 0.84 (-5.62;7.30) | 0.797 | 0.52 (-5,93;6.98) | 0.872 | -0.18 (-6.64;6.28) | 0.955 | -0.74 (-4.16;2.67) | 0.667 |
|  | Her2- & ER or PR+ vs. Triple - | -2.25 (-10.83;6.33) | 0.603 | -1.66 (-5.70;2.37) | 0.415 | -2.08 (-6.09;1.94) | 0.308 | -1.93 (-5.95;2.10) | 0.344 | **1.50 (-0.72;3.71)** | **0.183** |
| Theory of planned behavior | |  |  |  |  |  |  |  |  |  |  |
|  | **Beliefs about attending**  **≥ 30 sessions** |  |  |  |  |  |  |  |  |  |  |
|  | Intention | 1.71 (-1.93; 5.34) | 0.354 | 0.20 (-1.52;1.91) | 0.822 | -0.03 (-1.74;1.69) | 0.976 | 0.46 (-1.26;2.18) | 0.596 | NA | NA |
|  | Subjective Norm | 0.66 (-1.57; 2.88) | 0.559 | 0.32 (-0.73;1.36) | 0.550 | 0.24 (-0.81;1.28) | 0.656 | 0.58 (-0.46;1.62) | 0.269 | NA | NA |
|  | Perceived Behavioral Control | 0.37 (-2.58; 3.32) | 0.805 | 0.04 (-1.35;1.43) | 0.958 | 0.86 (-0.52;2.24) | 0.219 | 0.28 (-1.11;1.67) | 0.692 | NA | NA |
|  | Attitude | 0.18 (-2.73; 3.09) | 0.903 | 0.43 (-0.94;1.80) | 0.532 | 0.36 (-1.01;1.73) | 0.602 | 0.81 (-0.56;2.17) | 0.242 | NA | NA |
|  | **Beliefs about physical activity for ≥ 5 days per week** |  |  |  |  |  |  |  |  |  |  |
|  | Intention | NA | NA | NA | NA | NA | NA | NA | NA | **1.13 (0.31;1.95)** | **0.007** |
|  | Subjective Norm | NA | NA | NA | NA | NA | NA | NA | NA | **0.48 (-0.17;1.12)** | **0.147** |
|  | Perceived Behavioral Control | NA | NA | NA | NA | NA | NA | NA | NA | **0.57 (-0.14;1.28)** | **0.112** |
|  | Attitude | NA | NA | NA | NA | NA | NA | NA | NA | 0.27 (-0.38;0.92) | 0.410 |
| Psychological | |  |  |  |  |  |  |  |  |  |  |
| Anxiety | | -0.63 (-1.87; 0.61) | 0.318 | **-0.60 (-1.18;-0.03)** | **0.040** | -0.33 (-0.92;0.25) | 0.258 | **-0.53 (-1.10;0.05)** | **0.073** | 0.15 (-0.18;0.48) | 0.370 |
| Depression | | -0.79 (-2.14; 0.56) | 0.249 | **-0.69 (-1.31;-0.06)** | **0.031** | -0.39 (-1.02;0.24) | 0.225 | **-0.41 (-1.05;0.22)** | **0.197** | 0.01 (-0.35;0.37) | 0.948 |
| Health-related quality of life | | **0.18 (-0.03; 0.38)** | **0.090** | **0.11 (0.02;0.21)** | **0.018** | **0.07 (-0.02;0.17)** | **0.134** | **0.09 (-0.01;0.18)** | **0.070** | 0.01 (-0.05;0.06) | 0.876 |
| Beliefs about self-efficacy | |  |  |  |  |  |  |  |  |  |  |
|  | Attending 30 sessions | -0.76 (-3.07; 1.56) | 0.517 | **-1.03 (-2.11;0.04)** | **0.058** | -0.15 (-1.24;0.94) | 0.788 | -0.57 (-1.66;0.51) | 0.295 | NA | NA |
|  | Physical activity (≥5 days/week) | NA | NA | NA | NA | NA | NA | NA | NA | **0.39 (-0.18;0.96)** | **0.174** |
| Physical | |  |  |  |  |  |  |  |  |  |  |
| BMI (kg/m^2^) | | **-1.77 (-2.67; -0.87)** | **0.000** | **-0.58 (-1.02;-0.14)** | **0.011** | **-0.40 (-0.85;0.05)** | **0.079** | **-0.37 (-0.82;0.08)** | **0.108** | -0.11 (-0.38;0.16) | 0.434 |
| Baseline physical activity | | **0.01 (0.00; 0.02)** | **0.146** | 0.00 (0.00;0.01) | 0.202 | **0.00 (0.00;0.01)** | **0.072** | **0.00 (0.00;0.01)** | **0.069** | **0.00 (0.00;0.01)** | **0.005** |
| Physical Fatigue | | **-0.96 (-1,9; -0.01)** | **0.048** | **-0.51 (-0.96;-0.07)** | **0.024** | **-0.51 (-0.95;-0.07)** | **0.025** | **-0.39 (-0.84;0.07)** | **0.093** | -0.07 (-0.33;0.18) | 0.580 |
| Peak O_2_ Consumption (ml/min) | | 7.19 (-5.77; 20.14) | 0.273 | **6.85 (0.89;12.81)** | **0.025** | **6.71 (0.81;12.61)** | **0.026** | 1.38 (-4.72;7.48) | 0.654 | **4.36 (1.21;7.52)** | **0.007** |

**S2 Table**: **Univariate linear regression analyses on the association between the predictors and attendance and compliance.**

Bold is significant (p<0.2); Estimated unstandardized regression coefficients with 95% Confidence intervals are mentioned in this table. NA: not applicable
