# Supplementary material for: Attendance and compliance with an exercise program during localized breast cancer treatment in a randomized controlled trial: The PACT study
Source: PLoS One. 2019 May 8;14(5):e0215517. doi: 10.1371/journal.pone.0215517 (PMC6505930; doi:10.1371/journal.pone.0215517)
Supplement: S1 File — (PDF) [file pone.0215517.s003.pdf]

Physical Activity during Cancer Treatment (PACT) Study: a randomised clinical trial of physical exercise during cancer treatment

(December 2010)

Research protocol

Physical Activity during Cancer Treatment (PACT) Study: a randomised clinical trial of physical exercise during cancer treatment

**Protocol ID**	**PACT METC/005 (07/271)**
**Short title**	**Physical Activity during Cancer Treatment (PACT) Study: a randomised clinical trial of physical exercise during cancer treatment**
**Version**	**4**
**Date**	**December 2010**
**Coordinating investigator/project leader**	***Dr. P.H.M. Peeters***<br>***University Medical Center Utrecht***<br>***Julius Center***<br>***PO Box 85500***<br>***3508 GA Utrecht***<br>***T: 088-7559363***<br>***E: P.H.M.Peeters@umcutrecht.nl***
**Principal investigator(s) (in Dutch: hoofdonderzoeker/uitvoerder)**<br>***Multicenter research: per site***	***R. Koppejan-Rensenbrink, MSc***<br>***Comprehensive Cancer Center Middle Netherlands***<br>***PO Box 19079***<br>***3501 DB Utrecht***<br>***The Netherlands***<br>***T: 030-2338060***<br>***E: rkoppejan@ikmn.nl***<br><br>***M.J. Velthuis, MSc***<br>***Comprehensive Cancer Center Middle Netherlands***<br>***PO Box 19079***

3501 DB Utrecht

The Netherlands

T: 030-2337091

E: mvelthuis@ikmn.nl

Prof. Dr. E. van der Wall

University Medical Center Utrecht

DIGD, G02.228
PO Box 85500

3508 GA Utrecht

T: 088-7557409 / 7399
E: E.vanderWall@umcutrecht.nl

Prof. Dr. E. Lindeman

Rehabilitation center De Hoogstraat

PO Box 85238

3508 AE Utrecht

The Netherlands

T: 088-7559210

E: E.Lindeman@umcutrecht.nl

Dr. J.P. van den Berg

Meander Medical Center

PO Box 1502

3800 BM Amersfoort

The Netherlands

Drs. JE. Rütter

	Rivierenland Hospital<br>***PO Box 6024***<br>***4000 HA Tiel***<br>***The Netherlands***<br><br>***Dr. M. Los***<br>***Sint Antonius Hospital***<br>***PO Box 2500***<br>***3430 EM Nieuwegein***<br>***The Netherlands***<br><br>***Drs. D. ten Bokkel Huinink***<br>***Diakonessenhuis Utrecht***<br>***Postbus 80250***<br>***3508 TG UTRECHT***<br>***The Netherlands***
Sponsor (in Dutch: verrichter/opdrachtgever)	***UMC Utrecht, division Julius Center for Health Sciences and Primary Care and Comprehensive Cancer Center Middle Netherlands***
**Independent physician(s)**	***Dr. A. de Graeff***<br>***University Medical Center Utrecht***<br>***DIGD***<br>***PO Box 85500***<br>***3508 GA Utrecht***<br>***T:* 030-2506265**<br>***E: A.deGraeff@umcutrecht.nl***
**Laboratory sites**	***University Medical Center Utrecht***

PO Box 85500

3508 GA Utrecht

The Netherlands

Rehabilitation center De Hoogstraat

PO Box 85238

3508 AE Utrecht

The Netherlands

Meander Medical Center

PO Box 1502

3800 BM Amersfoort

The Netherlands

Rivierenland Hospital

PO Box 6024

4000 HA Tiel

The Netherlands

Sint Antonius Hospital

PO Box 2500

3430 EM Nieuwegein

The Netherlands

PROTOCOL SIGNATURE SHEET

Name	Signature	Date
**Head of Department:**<br>***Prof. Dr. A.W. Hoes, MD PhD***<br>***Julius Center for Health Sciences and Primary Care***<br>***University Medical Center Utrecht***		
**Coordinating Investigator/Project leader/Principal Investigator:**<br>***Dr. P.H.M. Peeters, Senior Epidemiologist***<br>***Julius Center for Health Sciences and Primary Care***<br>***University Medical Center Utrecht***		

TABLE OF CONTENTS

1. INTRODUCTION AND RATIONALE 12
2. OBJECTIVES 15
3. STUDY DESIGN 16
4. STUDY POPULATION 17
4.1 Population (base) 17
4.2 Inclusion criteria 17
4.3 Sample size calculation 17
5. INTERVENTIONS 18
5.1 Intervention 18
5.2 Control 19
6. METHODS 20
6.1 Study parameters/endpoints 20
6.1.1 Main study parameter 20
6.1.2 Secondary study parameters 20
6.1.3 Physical activity level 20
6.2 Randomisation, blinding and treatment allocation 20
6.3 Study procedures 21
6.3.1 Baseline measurements 21
6.3.2 Primary outcome measurements 21
6.3.3 Secondary outcome measurements 22
6.3.4 Physical activity level 26
6.4 Withdrawal of individual subjects 26
6.4.1. Specific criteria for withdrawal (if applicable) 26
6.5 Replacement of individual subjects after withdrawal 27
6.6 Follow-up of subjects withdrawn from treatment 27
6.7 Premature termination of the study 27
7. SAFETY REPORTING 28
7.1 Section 10 WMO event 28
7.2 Adverse and serious adverse events 28
7.2.1 Suspected unexpected serious adverse reactions (SUSAR) 29
7.2.2 Annual safety report 29
7.3 Follow-up of adverse events 29
7.4 Data Safety Monitoring Board (DSMB) 29
8. STATISTICAL ANALYSIS 30
8.1 Descriptive statistics 30
8.2 Primary analysis 30
9. ETHICAL CONSIDERATIONS 31
9.1 Regulation statement 31
9.2 Recruitment and consent 31
9.3 Objection by minors or incapacitated subjects (if applicable) 32
9.4 Benefits and risks assessment, group relatedness 32

9.5 Compensation for injury 32
9.6 Incentives..33
10. ADMINISTRATIVE ASPECTS AND PUBLICATION 34
10.1 Handling and storage of data and documents 34
10.2 Amendments..34
10.3 Annual progress report..34
10.4 End of study report...34
10.5 Public disclosure and publication policy 35
11. REFERENCES 36

LIST OF ABBREVIATIONS AND RELEVANT DEFINITIONS

ABR	ABR form (General Assessment and Registration form) is the application form that is required for submission to the accredited Ethics Committee (ABR = Algemene Beoordeling en Registratie)
AE	Adverse Event
AR	Adverse Reaction
CA	Competent Authority
CCMO	Central Committee on Research Involving Human Subjects
CV	Curriculum Vitae
DSMB	Data Safety Monitoring Board
EU	European Union
EudraCT	European drug regulatory affairs Clinical Trials GCP Good Clinical Practice
IB	Investigator's Brochure
IC	Informed Consent
IMP	Investigational Medicinal Product
IMPD	Investigational Medicinal Product Dossier
METC	Medical research ethics committee (MREC); in Dutch: medisch ethische toetsing commissie (METC)
(S)AE	Serious Adverse Event
SPC	Summary of Product Characteristics (in Dutch: officiële productinfomatie IB1-tekst)
Sponsor	The sponsor is the party that commissions the organisation or performance of the research, for example a pharmaceutical company, academic hospital, scientific organisation or investigator. A party that provides funding for a study but does not commission it is not regarded as the sponsor, but referred to as a subsidising party.
SUSAR	Suspected Unexpected Serious Adverse Reaction
Wbp	Personal Data Protection Act (in Dutch: Wet Bescherming Persoonsgevens)
WMO	Medical Research Involving Human Subjects Act (Wet Medisch-wetenschappelijk Onderzoek met Mensen

SUMMARY

Rationale: Cancer related fatigue is one of the major problems of cancer patients. Sixty% - 96% of the cancer patients report high levels of fatigue during or after cancer treatment (1, 2). We hypothesize that early physical exercise will increase physical fitness and thereby diminish complaints of fatigue. The effect will be during the exercise program, but will also affect health after longer term. This may result in a decrease of healthcare related expenditures by reducing healthcare utilization.

Objective: The objective of the purposed randomised controlled clinical trial is to answer the following research questions:

1) Is a 18 week groupwise supervised exercise program during cancer treatment effective in reducing complaints of fatigue (primary outcome) and health service utilization and improving health related quality of life, physical fitness, body composition, cognitive-behavioral aspects and biomarkers that are hypothesized to be in the causal pathway between physical exercise and reduction of fatigue (inflammation markers and haemoglobin) and in the causal pathway between physical activity and cancer risk (inflammation markers, sex steroid hormones, and insulin) (secondary outcomes)?.
    a. Short term effects will be measured after 18 weeks
    b. Long term effects will be measured after 9 months
2) What is the cost-effectiveness of a 18 week groupwise supervised exercise program during cancer treatment?

Study design: A randomised controlled clinical study.

Study population: A total of 342 newly diagnosed patients with breast or colon cancer (stage M0) admitted for (adjuvant) cancer treatment will be recruited.

Intervention: The intervention group (n=171) will receive an 18 week supervised group exercise program based on the theory of planned behavior during cancer treatment. The control group will receive care as usual (no exercise program).

Main study parameters/endpoints: The exercise program will start at the same time as the start of the (adjuvant) cancer treatment. It is supposed to prevent or diminish complaints of fatigue and to decrease health service utilization.

Primary outcome measure: fatigue

Secondary outcome measures: health service utilization, health related quality of life (general quality of life, functional well being, emotional well being), physical fitness (muscle strength, aerobic capacity), body composition (body mass index, body fat distribution), biomarkers (inflammation markers, sex steroid hormones, insulin, and haemoglobin) and cognitive-behavioral aspects (behavior intentions, perceived behavioral control, self efficacy).

Nature and extent of the burden and risks associated with participation, benefit and group relatedness:

The patient will be asked three times to spend an hour at completing questionnaires, providing a blood sample and performing several physical tests in a period of 9 months (baseline measurement and two follow up measurements). In addition patients will be asked to keep a log in which they register activity levels and health service utilization during the 9-months study period. Completing questionnaires and physical tests and keeping a log can confront the patient with the consequences of his/ her disease which may be experienced as a psychological burden. Patients allocated to the intervention group are supposed to participate in an 18 –week group exercise program of 2 hours a week. The estimated extra risk for the patient while participating in this study is low. We expect that the exercise program will have a beneficial effect on the patients' health status. During the exercise program patients will meet fellow sufferers, which may also be beneficial for their psychological health. We tried to reduce the burden of travelling to the training facilities by offering the exercise program on five locations in the region Middle Netherlands (Utrecht (2x), Baarn, Nieuwegein and Tiel). Blood sampling is a routine that may, however, cause mild bruising or a haematoma around the area where the needle went into the vein.

1. INTRODUCTION AND RATIONALE

Healthcare problem

In the Netherlands, almost 70.000 persons - 0.5% of the Dutch population - are newly diagnosed with cancer each year. About 50% of the cancer patients survive more then five years. This results in a high number of cancer survivors - 366.000 - which will increase due to increase of the elderly population (3). Special attention for health care of cancer patients during diagnosis, treatment and follow up is necessary. The Dutch Health Council recently published a report about the follow up of cancer patients (4). They plead for more evidence based interventions for cancer patients during treatment. This study aims at studying this with special emphasis on physical fitness.

Subject of this proposal

One of the major problems of cancer patients is fatigue. Sixty% - 96% of the cancer patients report high levels of fatigue during or after cancer treatment (1, 2). Fatigue is also affecting quality of life. The hypothetical determinants of fatigue are i.e. direct effects of cancer and the effects of cancer treatment (1). Deconditioning is thought to contribute also to increased levels of fatigue. Additional reduction of physical activity levels in patients diagnosed with cancer will further affect fatigue (2, 5) (see figure 1).

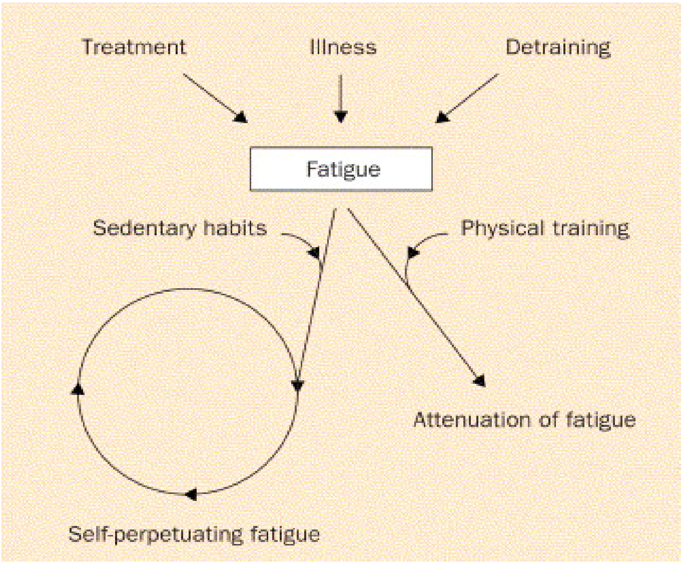

Figure 1: Causes of fatigue (Lucia 2003)

The National Comprehensive Cancer Network, an alliance of 21 of the world's leading cancer centres, already advises to start physical exercise shortly after the cancer diagnosis (6). In line with this advice we would like to establish the effects of early physical exercise. By doing so best evidence will be generated for this health care intervention. We hypothesize that early physical exercise will diminish a decrease in physical fitness and complaints of fatigue. The effect will be during the exercise program, but will also affect health after longer term. The care of cancer patients will improve by reducing fatigue, the most frequently reported symptom. Decrease of complaints of fatigue may also lead to an improvement of quality of life. Altogether this may result in a decrease of healthcare related expenditures by reducing healthcare utilization.

Effectiveness of physical exercise during cancer treatment on fatigue and health related quality of life has been studied before. Most studies after physical exercise during cancer treatment were done in breast cancer patients. Two reviews studied the effects of physical exercise during cancer treatment in breast cancer patients (7, 8). One review showed that physical exercise during treatment improves fatigue, quality of life and physical fitness (8). The other showed that physical exercise positively affects physical fitness, although improvements of fatigue were ambiguous (8).

Fewer and methodologically more limited studies have been performed in other cancer patients. Physical exercise during treatment was studied in leukaemia, stomach, prostate and mixed cancer populations. These reviews showed unambiguous that physical exercise is effective to improve physical fitness (9-11). They show positive trends in improvements of fatigue and quality of life in mixed cancer populations (9-11).

In the Netherlands, effectiveness of physical exercise during cancer treatment has not been studied before. On the other hand, several non- randomised trials have described exercise programs after cancer treatment (12-15). They showed improvements in quality of life of cancer survivors after participating in these exercise programs (12-15). These studies were followed by a KWF-funded RCT studying the effects of physical exercise after cancer treatment. The results of this so called ONCOREV study have not been published yet.

Markes described that the little evidence that cancer patients may benefit from physical exercise during cancer treatment may possibly be caused by adherence problems and the poor evaluation of adherence and the extent to which the control group performs exercise (8). Adherence to an increased physical exercise level is a major challenge for cancer

patients which require them to change their behavior. Markes suggested that in order to occur a behavior change – adherence to exercise (program) – it is essential that exercise programmes focus on underlying principles from theories about why people change their behaviors (9). The theory of planned behavior has frequently been used to understand the determinants of exercise behavior in cancer patients and survivors (16-18). Central to the theory of planned behavior is the idea that performance of behavior – adherence to an increased physical exercise level - is determined by behavioral intentions. Behavioral intentions are representations of people's plans of action which summarize people's motivation to engage in behavior (19). Behavioral intentions are influenced by perceived behavioral control, subjective norm (perceived social pressure to perform behavior) and attitude (positive-negative evaluations of behavior). Perceived behavioral control reflects' peoples confidence to carry out a particular behavior. Jones et al studied the effectiveness of an exercise recommendation based on the theory of planned behavior and suggested that interventions must change perceived behavioral control in order to change exercise behavior (20).

The aim of this study is to examine the effectiveness of a groupwise supervised exercise program during cancer treatment. The idea is that this exercise program is offered to cancer patients much earlier in time as compared to regular programs nowadays in the Netherlands that are offered to cancer patients, starting on average two – three months after cancer treatment has finished. Usually 12 -18 months after diagnosis. This exercise program will start before the start of the adjuvant cancer treatment. To stimulate participant's adherence to an increased physical exercise level, it will be based on the theory of planned behaviour (19).

OBJECTIVES

The objective of this study is to answer the following research questions:

1) Is a 18 week groupwise supervised exercise program during cancer treatment effective in reducing complaints of fatigue (primary outcome) and health service utilization and improving health related quality of life, physical fitness, body composition, cognitive-behavioral aspects and biomarkers that are hypothesized to be in the causal pathway between physical exercise and reduction of fatigue (inflammation markers and haemoglobin) and in the causal pathway between physical activity and cancer risk (inflammation markers, sex steroid hormones, and insulin) (secondary outcomes).
    a. Short term effects will be measured after 18 weeks
    b. Long term effects will be measured after 9 months
2) What is the cost-effectiveness of a 18 week groupwise supervised exercise program during cancer treatment?

Before the start of this study we planned a pilot study (start expected in May 2008). The design of the pilot study is comparable to the design to this study which will be described in the following paragraphs. The pilot study will include 42 cancer patients (breast, colon and prostate) at the SFC De Hoogstraat in Utrecht. This pilot studies aims to study the feasibility of the exercise program in cancer patients. We will investigate whether it is possible to include 25 cancer patients (including breast, colon and prostate) per month in the PACT-study. Furthermore we will examine the compliance to the study and to the exercise program. We expect that 80-90% of the participants will attend the measurements at baseline and after 18 and 52 weeks. We expect that 60-70% of the participants will participate in at least 70% of the exercise sessions. After the pilot study the exercise program will be evaluated with physiotherapists and patients, as a part of the process of Intervention Mapping. Furthermore, the Zelen design will be evaluated with the medical specialists.

2. STUDY DESIGN

This study will be a randomised controlled clinical study. During the pilot study a Zelen-design randomisation was used to distribute patients to the supervised group exercise program during cancer treatment (intervention) or to usual care (control) (21). According to the Zelen design patients will be asked for consent to follow up and postponed information until the end of follow up. Subsequently patients will be randomly allocated to the intervention or control group. After randomisation those randomised to the intervention will be informed about the intervention. This design is chosen because patients allocated to the control group will likely be disappointed and may seek possibilities for exercise by themselves or withdraw from the study. This was clearly seen in the randomised study to measure effects of physical exercise after cancer treatment has finished the so called ONCOREV study.

The pilot study showed that the Zelen randomisation was not effective. Study subjects offered to participate in the exercise programme withdraw because of the unexpected confrontation with the programme. In addition many control women met the intervention group during chemotherapy and therefore knew about the exercise programme. Due to these findings, we decided to use conventional randomisation in the definitive study.

3. STUDY POPULATION

3.1 Population (base)

A total 342 newly diagnosed breast, or colon cancer patients (171 per cancer diagnosis) will be recruited by medical specialists or oncology nurses of collaborating hospitals in the region Middle Netherlands (Antonius hospital, Diakonessenhuis, Meander Medical Center, Mesos Medical Center, Diakonessenhuis Utrecht, University Medical Center Utrecht and Zuwe Hofpoort Woerden, Rivierenland hospital Tiel). All collaborating hospitals together treat annually about 1100 patients suffering from breast cancer and 800 patients suffering from colon cancer. So, we aim to finish our inclusion in 1.5 year.

The exercise program is targeted towards cancer patients who are expected to suffer from fatigue. The study population is restricted to the two most prevalent types of cancers: breast and colon (3). Patients included will have newly diagnosed cancer ((< 6 weeks) (no distant metastases (M0)) and will be treated with adjuvant regimens. Included will be breast and colon cancer patients who will be treated with chemotherapy. Included were men and women, aged between 25 and 75.

3.2 Inclusion criteria

Newly diagnosed breast and colon cancer patients (stage M0) will be eligible for this study. Breast and colon cancer patients are admitted for chemotherapy. Inclusion criteria are: a) definite diagnosis of cancer was less then six weeks ago; b) not previously treated for cancer (except basal skin cancer) c) age 25-75; d) able to read and understand the Dutch language; e) Karnovsky Performance Status of 60 or higher; f) able to walk 100 meter or more; g) not reporting contra indications for physical activity on the Revised Physical Activity Readiness Questionnaire (PARQ) (22)

3.3 Sample size calculation

To detect an intervention effect of 2 units of change (±SD 4) in fatigue (based on the Multidimensional Fatigue Inventory (range subscale 4-20)) we will need 75 participants in the intervention and control group of the RCT for each cancer diagnosis, 450 participants in total (p=0.05, 80% power). A drop out of 10% is included. Change is based on a descriptive study of physical exercise in cancer survivors although it is unknown if change is clinically relevant (13). However, to detect a clinically relevant intervention effect of 10 units of change (±SD 20) on the EORTC QLQ C30 the same sample size should be required (12).

4. INTERVENTIONS

4.1 Intervention

Participants who will be assigned to the intervention will be offered an 18 week supervised group exercise program. The exercise program is based on the theory of planned behavior. It will start earliest one week after surgery and at least before adjuvant cancer treatment, within six weeks after diagnosis (see Figure 2 and 3). Before the start of the exercise program program, but preferably after surgery, the patient will be asked to visit the trial center for an intake meeting, inclusion and randomisation. The exercise program is planned at four outpatient clinic or rehabilitation settings. Participants will be encouraged to attend two times a week the exercise program supervised by a physical therapist. Classes will be timetabled at various times in the day and evening.

Participants of the intervention group will be advised to be physically active for 30 minutes a day, five days a week (Nationale Norm voor Bewegen (Dutch guideline for physical activity)). This should include an aerobic component in agreement with participant's fitness and desires. Participants will be advised to perform aerobic training at a moderate intensity.

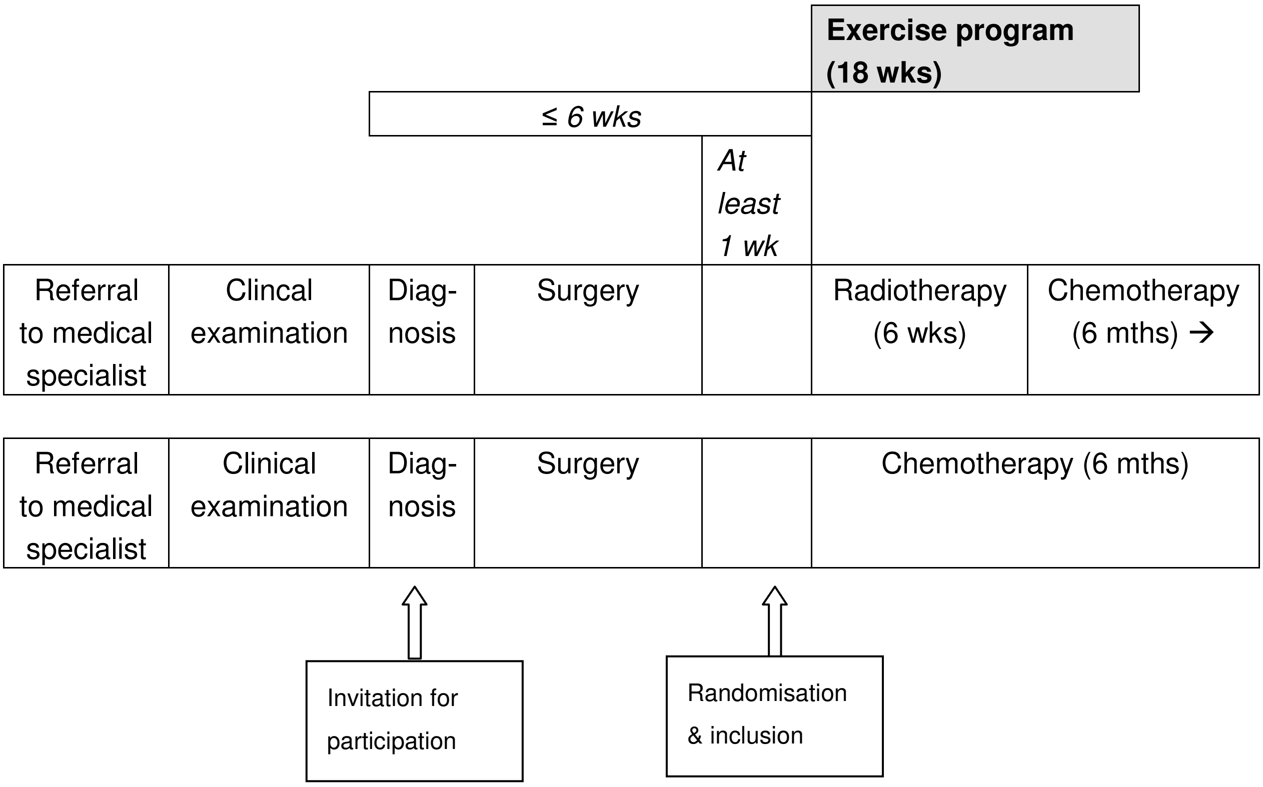

Figure 2: Treatment schedule breast and colon cancer

The exercise program will aim to realize a behavior change, to obtain increase of the physical exercise level. This will be accomplished by a cognitive behavioral intervention based on the theory of planned behaviour. Increase of the control beliefs should give rise to perceived behavioral control (19).

The program will be individualized to the patient's condition and personal preferences (23). Patients' condition will be determined during baseline exercise tests. Patient's personal preferences will be asked during an intake by the physiotherapists prior to the first exercise class. The 1-hour exercise classes will include a warming up (10 min), aerobic and muscle strength training (40 min) and cooling down (10 min). Aerobic training will be performed as interval training with an alternating intensity at and below the ventilatory threshold (3 x 2 min increasing to 2 x 7 min.) as determined during the baseline maximal exercise test. Heart rates and the Borg scale of perceived exertion will be monitored during the aerobic training. Muscle strength training will be performed for all major muscle groups – arms, legs, shoulders, and trunk: 2 x 10 repetitions (65% 1-RM) increasing to 1 x 10 repetitions (75% 1-RM) and 1 x 20 repetitions (45% 1-RM). Muscle strength training is based on 1-RM measurements

4.2 Control

The participants who will be assigned to the control group will receive care as usual. This includes an optional choice for Herstel & Balans. Herstel & Balans is a combination of a physical exercise and educational program that starts three months after cancer treatment has finished. Herstel & Balans started in 1997 and nowadays the program is offered at 55 locations in the Netherlands and Belgium. Participants allocated to the control group will be asked to visit the trial center three times during a one-year period to complete questionnaires and perform physical tests.

METHODS

4.3 Study parameters/endpoints

4.3.1 Main study parameter

The main study parameter is fatigue.

4.3.2 Secondary study parameters

The secondary study parameters are: health service utilization, health related quality of life (general quality of life, functional well being, emotional well being), physical fitness (muscle strength, aerobic capacity), body composition (body mass index, body fat distribution), cognitive-behavioral aspects (behavior intentions, perceived behavioral control, self efficacy), and biomarkers that are hypothesized to be in the causal pathway between physical exercise and reduction of fatigue (inflammation markers and haemoglobin) and in the causal pathway between physical activity and cancer risk (inflammation markers, sex steroid hormones, and insulin).

4.3.3 Physical activity level

In addition to the main and secondary study parameters the physical activity level will be assessed.

4.4 Randomisation, blinding and treatment allocation

Randomisation will be used to distribute patients to the supervised group exercise program during cancer treatment or to usual care. During the visit at the outpatient clinic the medical specialist or oncology nurse will invite newly diagnosed cancer patient to participate in the study. Patients who are willing to participate will be asked to announce within one week (by telephone) to the research assistant. The patient will be invited for an intake meeting by the research assistant at the trial center (University Medical Center Utrecht). The research assistant will determine if the patient is eligible for the study. Eligible patients will be asked for consent to follow up. After informed consent, patients will be randomly allocated per tumour site (breast or colorectal) to the intervention or the control group by the sequential balancing method. For balancing age will be included as (25-40, 40-65 and 65-75 years) as first step in the balancing algorithm, followed by adjuvant treatment (radiotherapy vs. no radiotherapy (before chemotherapy)), and gender in colorectal cancer patients. Prior to randomisation patients will be informed about the study aims. After randomisation patients in the intervention group will be informed about the supervised group exercise program and the exercise recommendation to be physically active for 30 minutes a day, five days a week. Patients in the control group will be informed about their control status. Baseline measures will be obtained.

4.5 Study procedures

At baseline, after 18 weeks and 9 months all participants will visit the research center for physical measurements and filling out questionnaires. Short term effects will be measured 18 weeks after inclusion (at the end of the program) and long term effects will be measured 9 months after inclusion. Throughout the whole study duration participants fill out diaries to assess their health care use and work status. Participants of the exercise group fill out exercise logs for the duration of the exercise program. Participants will be asked to wear a pedometer during seven days following both baseline and follow-up measurements.

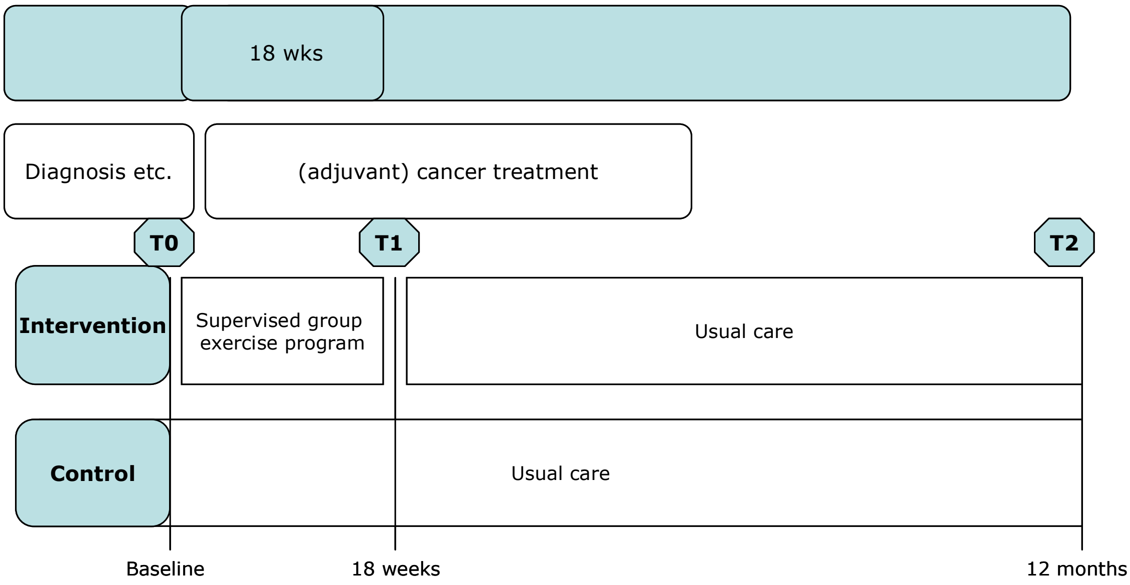

Figure 4: Overview of measurements

4.5.1 Baseline measurements

At baseline (T0, at randomisation) standard demographic data and factors predictive for fatigue will be noted. Clinical information regarding the treatment regime will be requested from medical specialist or oncology nurse.

4.5.2 Primary outcome measurements

6.3.2.1 Fatigue

At randomisation (T0), after 18 weeks (T1) and after 9 months (T2) fatigue will be assessed. Fatigue will be measured using the Multidimensional Fatigue Inventory (MFI) and the Fatigue Quality List (FQL), both questionnaires with adequate psychometric properties (25). The MFI is a 20-item self-report instrument designed to measure multiple fatigue characteristics and their impact on function (general fatigue, physical fatigue, reduced activity, reduced motivation and mental fatigue). Each item is scored in one of five categories, which ranges form 'totally agree' to 'not agree at all'. Higher scores indicate a higher degree of fatigue. The FQL is a list consisting of 28 adjectives addressed to assess the perception of fatigue [27]. Participants are asked to mark with a cross which of the 28 adjectives fit their experienced fatigue. Multiple answers are possible. The adjectives are clustered in four subscales: frustrating, exhausting, pleasant and frightening. Each subscale will be recoded to a 0-100 scale. Higher scores indicate a higher appraisal of the fatigue experience.

4.5.3 Secondary outcome measurements

The following secondary outcome measurements will be conducted at randomisation (T0), after 18 weeks (T1) and after 9 months (T2).

6.3.3.1. Health service utilization and sick leave

During the study period, participants will keep diaries to register visits or telephone calls to health services. These diaries will contain all categories of health care consumption that are prominent in cancer patients, including alternative medicine and own out of pocket expenses. Participants will be asked to keep track of their absence from work (if applicable) during the whole period of intervention and follow-up.

The diaries include also the EQ-5D , a multidimensional measurement of health. The EQ-5D will be used to calculate quality adjusted life years (QALYs) and consists of the EQ-5D descriptive system and the EQ VAS [28]. The EQ-5D descriptive system comprises 5 dimensions of health (mobility, self-care, usual activities, pain/discomfort anxiety/depression). Each dimension comprises three levels (no problems, some/moderate problems/extreme

problems). The EQ VAS records the respondents self-rated health status on a vertical graduated (0-100) visual analogue scale.

6.3.3.2. Health related quality of life

To measure health related quality of life the EORTC-QOL-C30 questionnaire (version 3) and the Short Form 36 healthy survey (SF-36) will be used. Both questionnaires have been validated [29-31].

The EORTC QOL C30 incorporates five functional scales (1) physical functioning (two items), 2) role functioning (two items), 3) emotional functioning (four items), 4) cognitive functioning (two items) and 5) social functioning (two items)), one quality of life scale (two items) and one symptom scale (13 items, including fatigue (three items) and pain (two items)). Each item is scored in one of four categories (not at all, a little, quite a bit and very much), with the exception of 'quality of life' which ranges from 1-7 (Very poor to Excellent). After recoding and transformation the total score ranges from 0 to 100: a higher score represents a higher level of functioning, or a higher level of symptoms.

The SF-36 consists of 36 items, organised into eight scales: physical functioning, role limitations due to physical health problems, bodily pain, general health perceptions, vitality, social functioning, role limitations due to emotional problems and general mental health. All scales will be converted linearly to a 0-100 scale, with higher scores indicating better functioning of well-being.

Impact on Participation and Autonomy

The perceived personal impact of the disease on participation and autonomy will be measured with the validated Impact on Participation and Autonomy (IPA) questionnaire [32,33]. The IPA incorporates 32 items clustered in the subscales autonomy indoors (7 items), family role (7 items), autonomy outdoors (5 items), social life and

relationships (7 items) and work and education (6 items). IPA items have five response categories ('very good', 'good', 'fair', 'poor', 'very poor').

Anxiety and Depression

Anxiety and depression will be self-rated with the Dutch language version of the Hospitality Anxiety and Depression Scale (HADS) (28). The HADS consists of 14 items, seven items of the depression subscale (HADS-D) and seven items of the anxiety subscale (HADS-A). Items will be scored on a 4-point Likert scale. Depression or anxiety disorders were defined by a score of greater than or equal to 8 on HADS-D or HADS-A respectively.

6.3.3.3. Physical fitness

Muscle strength

Muscle strength of quadriceps and hamstring will be assessed by using a Cybex II dynamometer (Lumex, Ronkonkoma, NY) at 60°/s and 180°/s. A standardized 5-minute warm-up will be performed before Cybex testing. Five repetitions will be performed for practice before the definitive measurements at 60°/s and 180°/s will be taken. Between all sessions, there will be a 1-minute rest period and the patient will be verbally encouraged. The highest peak torque value for each velocity will be calculated.

Handgrip strength will be measured with a mechanical handgrip dynamometer. The best score of five attempts will be recorded in kilogram force (kgF).

Aerobic capacity

Aerobic capacity will be assessed using a symptom-limited bicycle ergometry test using a ramp 10-, 15-, or 20-protocol, dependent on the patients condition. The load will be increased every minute by 10, 15, or 20 Watts respectively, in such a way that patients will reach their maximal workload within 10 minutes. The test will be terminated on the patients' symptoms or at the physicians discretion. Borg scores for dyspnoea and muscle fatigue will be taken before and after the test. Maximal workload, maximal oxygen uptake and Borg scores at maximal workload will be taken for analysis.

6.3.3.4. Body Composition

Body Mass Index

The Body Mass Index (BMI) will be calculated as weight in kilograms divided by height in meters squared (kg/m^2). Body weight and height (to the nearest 0.5 kg and 0.5 cm respectively) will be measured while the subjects wear light clothes and no shoes using an analogue balance (SECA) and wall mounted tape measure.

Body fat distribution

Body fat distribution will be measured by the waist- and hip circumference. Waist circumference (to the nearest 0.1 cm) will be measured standing at the smallest circumference between abdomen and chest. Hip circumference (to the nearest 0.1 cm) will be measured standing as the largest circumference between waist and thigh. All measurements will be taken in duplicate and averaged

6.3.3.4. Cognitive behavioral aspects

Behavior intentions

Four items based on the Theory of Planned Behavior will be used to assess the intentions of to be physically active for 30 minutes during at least five days per week (30). Responses were recorded on 5-point Likert scales with endpoints labelled ‘strongly disagree’ and ‘strongly agree’.

Perceived behavioral control

Perceived behavioral control will be assessed by six items based upon the general recommendations of the Theory of Planned Behavior developers (30). Items like ‘If I wanted I could be physically active during at least five days a week’ will be used. Items will be scored on a 5-point Likert scale with endpoints labelled ‘strongly disagree’ and ‘strongly agree’.

Self Efficacy

Self efficacy will be assessed by six items based upon the Social Cognitive Theory (31). Items will be scored on a 5-point Likert scale with endpoints labelled ‘strongly disagree’ and ‘strongly agree’.

6.3.3.5. Blood samples

Blood samples will be drawn at randomisation (T0), after 18 weeks (T1) and after 9 months (T2) in order to determine serum concentrations of inflammation markers (IL-1 beta, IL-4, IL-

6, IL-8, IL-10, TNF alpha and CRP), sex steroid hormones, insulin and hemoglobin. At each sampling time blood will be taken at rest (30 mL) after a 2-hour fasting period. Blood samples will be taken at the same time of the day for each participant. Participants will be asked to refrain from physical exercise the preceding 24 hours. Blood samples will be stored at -70°C until analyses. All samples from one individual will be analysed in the same batch. Blood analyses will be done at the Institute for Risk Assessment Sciences (IRAS, University of Utrecht) using the Luminex technology.
Note, that the blood sampling will be a voluntary additional measurement to the PACT study for which extra consent will be asked. Participants can withdraw the blood taking without withdrawing from the study.

4.5.4 Physical activity level

Physical activity level will be measured using the Short Questionnaire to assess health enhancing physical activity (SQUASH).
In addition, participants allocated to the intervention group will be asked to keep an exercise log. In the log they register the frequency, intensity, and duration of the exercises they were performing during the study period. In addition physical activity will be measured by a pedometer. Participants will wear the pedometer during seven weekdays following their baseline or follow up measurement. The pedometer will be placed on the belt or waistband, approximately five to seven centimetres from the umbilicus. The stride length will be determined to compute distances walked. To determine stride length participants will be asked to take 20 strides at their normal walking pace. The distance walked will be divided by 20 to determine the average stride length, which will be entered into the pedometer (32).

Adherence to the exercise programme
Attendance rate for the exercise sessions will be recorded in a Case Record Form.
Adherence to the exercise recommendation will be registered in the exercise log.

4.6 Withdrawal of individual subjects

Subjects can leave the study at any time for any reason if they wish to do so without any consequences. The investigator can decide to withdraw a subject from the study for urgent medical reasons.

6.4.1. Specific criteria for withdrawal (if applicable)

Not applicable

4.7 Replacement of individual subjects after withdrawal

Patients withdrawing from the study will not be replaced.

4.8 Follow-up of subjects withdrawn from treatment

Patients withdrawing from the study will be asked to show up for the follow up measurements.

4.9 Premature termination of the study

The study will be terminated prematurely when an insufficient number of patients can be recruited or when the intervention or testing procedures are not tolerated by the patients. Based on previous studies and Herstel & Balans we do not expect this will be the case.

5. SAFETY REPORTING

5.1 Section 10 WMO event

In accordance to section 10, subsection 1, of the WMO, the investigator will inform the subjects and the reviewing accredited METC if anything occurs, on the basis of which it appears that the disadvantages of participation may be significantly greater than was foreseen in the research proposal. The study will be suspended pending further review by the accredited METC, except insofar as suspension would jeopardise the subjects' health. The investigator will take care that all subjects are kept informed.

5.2 Adverse and serious adverse events

Usual procedures are in place safety during the exercise program and exercise testing. This means that the exercise program will be supervised by a physiotherapist. One physiotherapist will be responsible for maximal six patients. Testing will take place under supervision of experienced staff; a medical specialist will be available in case of emergencies.

All adverse events reported spontaneously by the subject or observed by the investigator or his staff will be recorded.

A serious adverse event is any untoward medical occurrence or effect that at any dose results in death;

- is life threatening (at the time of the event);
- requires hospitalisation or prolongation of existing inpatients' hospitalisation;
- results in persistent or significant disability or incapacity;
- is a congenital anomaly or birth defect;
- is a new event of the trial likely to affect the safety of the subjects, such as an unexpected outcome of an adverse reaction, lack of efficacy of an IMP used for the treatment of a life threatening disease, major safety finding from a newly completed animal study, etc.

All SAEs will be reported to the accredited METC that approved the protocol, according to the requirements of that METC.

5.2.1 Suspected unexpected serious adverse reactions (SUSAR)

Not applicable

5.2.2 Annual safety report

Not applicable

5.3 Follow-up of adverse events

All adverse events will be followed until they have abated, or until a stable situation has been reached. Depending on the event, follow up may require additional tests or medical procedures as indicated, and/or referral to the general physician or a medical specialist.

5.4 Data Safety Monitoring Board (DSMB)

Not applicable

6. DATA ANALYSIS AND ECONOMIC EVALUATION

6.1 Descriptive statistics

Descriptive statistics will be used to describe the study population and the instrument scores at baseline and at the follow up measurements. Mean differences including 95% confidence intervals will be calculated. Mean differences will be presented for the total study population and for different subgroups (cancer diagnosis, cancer treatment etc.).

6.2 Primary data analysis

The primary analysis will test whether significant differences exist between change scores in the intervention and control group between baseline and after 18 weeks and nine months. Comparisons between intervention and control group will be determined using unpaired t-tests for interval and ratio data and using the Mann Whitney U test for ordinal data. If a meaningful difference occurs between both groups in baseline characteristics regression analyses will be used for adjustment. Analysis will be performed on an intention to treat and a per protocol basis. Statistical analysis will be performed using the Statistical Package for the Social Sciences (SPSS for Windows, release 14.0; SPSS Inc., Chicago, Illinois, USA)

6.3 Economic evaluation

The aim of the economic evaluation is to assess the balance between costs and effects of a supervised exercise program versus no intervention in cancer patients during treatment. The initial approach is to compare the actual costs incurred with both strategies up until 9 months after randomisation. Costs estimates will be based on the actual costs – direct and indirect – in both study arms. Direct costs include the exercise program (estimated on €500 per person). Indirect costs include costs of health service utilization associated with the cancer. Effects related to loss of quality of life and higher rate of complaints of fatigue in the control group will be estimated and result in estimates of 'gained QALYS' and in terms of years in 'good health' in the intervention group. Sixty to 96% of the annually 70.000 newly diagnosed cancer patients suffer from fatigue. By offering a physical exercise program during cancer treatment fatigue is hypothesized to reduce by 40%-50%.

Incremental cost-effectiveness ratios (ICER) will be generated by calculating the incremental costs of the exercise program compared to the non-intervention group divided by the incremental effects. Ultimately incremental costs per year in 'good health' gained will be estimated with a one year time horizon.

7. ETHICAL CONSIDERATIONS

7.1 Regulation statement

This study will be conducted according to the principles of the Declaration of Helsinki version 52, adapted in Tokyo and in accordance with the Medical Research Involving Human Subjects Act (WMO).

7.2 Recruitment and consent

A Zelen-design randomisation (single consent method) will be used to distribute patients to the supervised group exercise program during cancer treatment or to usual care (21). Zelen's design will be used, because we believe that patients allocated to usual care will be disappointed and will seek physical activity by their own. This was clearly seen in the randomised study to measure effects of physical exercise after cancer treatment has finished the so called ONCOREV study.

During the visit at the outpatient clinic the medical specialist or oncology nurse will invite newly diagnosed cancer patient to participate in the study. Patients will also be asked for permission to call them, if they have not announced within one week. Patients who are willing to participate will be asked to announce within one week (by telephone) to the research assistant. Subsequently the patient will be invited for an intake meeting by the research assistant at the trial center (University Medical Center Utrecht). The research assistant will determine if the patient is eligible for the study. Eligible patients will be asked for consent to follow up. After informed consent, patients will be randomly allocated to the intervention or control group per tumour site (breast or colorectal) to the intervention or the control group by the sequential balancing method. For balancing age will be included as (25-40, 40-65 and 65-75 years) as first step in the balancing algorithm, followed by adjuvant treatment (radiotherapy vs. no radiotherapy (before chemotherapy)), and gender in colorectal cancer patients. Prior to randomisation patients will be informed about the study aims. After randomisation patients in the intervention group will be informed about the supervised group exercise program and the exercise recommendation to be physically active for 30 minutes a day, five days a week. Patients in the control group will be informed about their control status.

Note, that the blood sampling will be a voluntary additional measurement to the PACT study for which extra consent will be asked. Participants can withdraw the blood taking without withdrawing from the study.

7.3 Objection by minors or incapacitated subjects (if applicable)

Not applicable

7.4 Benefits and risks assessment, group relatedness

Patients allocated to both the intervention and the control group will be asked three times to spend an hour at completing questionnaires and performing several physical tests in a period of 9 months (baseline measurement and two follow up measurements). In addition all patients will be asked to keep a log in which they register activity levels and health service utilization during the 9-months study period. This can confront him of her with the consequences of his/ her disease which may be experienced as a psychological burden.

Patients allocated to the intervention group are supposed to participate in a 18 –week group exercise program of 2 hours a week. The estimated extra risk for the patient while participating in this study is low. We expect that the exercise program will actually have a beneficial effect on the patients' health status. We hypothesize that early physical exercise will diminish a decrease in physical fitness and complaints of fatigue. The effect will be during the exercise program, but will also affect health after longer term. The care of cancer patients will improve by reducing fatigue, the most frequently reported symptom. Decrease of complaints of fatigue may also lead to an improvement of quality of life. Thereby, patients will meet fellow sufferers during the exercise program, which may also be beneficial for their psychological health. We tried to reduce the burden of travelling to the training facilities by offering the exercise program on five locations in the region Middle Netherlands (Utrecht (2x), Baarn, Nieuwegein and Tiel).

7.5 Compensation for injury

The sponsor/investigator has a liability insurance which is in accordance with article 7, subsection 6 of the WMO.

The sponsor (also) has an insurance which is in accordance with the legal requirements in the Netherlands (Article 7 WMO and the Measure regarding Compulsory Insurance for Clinical Research in Humans of 23th June 2003). This insurance provides cover for damage to research subjects through injury or death caused by the study.

1. € 450.000,-- (i.e. four hundred and fifty thousand Euro) for death or injury for each subject who participates in the Research;
2. € 3.500.000,-- (i.e. three million five hundred thousand Euro) for death or injury for all subjects who participate in the Research;

3. € 5.000.000,-- (i.e. five million Euro) for the total damage incurred by the organisation for all damage disclosed by scientific research for the Sponsor as ‘verrichter’ in the meaning of said Act in each year of insurance coverage.

The insurance applies to the damage that becomes apparent during the study or within 4 years after the end of the study.

7.6 Incentives

Through participation in the study participants allocated to the intervention group will be offered a 18- week supervised group exercise program (twice a week). This gives them also the opportunity to share experiences with fellow sufferers. Participants allocated to the intervention group will receive an advice to be physically active for 30 minutes a day, five days a week.

8. ADMINISTRATIVE ASPECTS AND PUBLICATION

8.1 Handling and storage of data and documents

Data are coded anonymous. Data check and entry into the computer is done by the trial center of the Comprehensive Cancer Center Middle Netherlands. Access to names, without access to the database is only allowed for principal investigators. All people working at the study signed for Good Clinical Practice (GCP).

8.2 Amendments

Amendments are changes made to the research after a favourable opinion by the accredited METC has been given. All amendments will be notified to the METC that gave a favourable opinion.

All substantial amendments will be notified to the METC and to the competent authority.

Non-substantial amendments will not be notified to the accredited METC and the competent authority, but will be recorded and filed.

8.3 Annual progress report

The investigator will submit a summary of the progress of the trial to the accredited METC once a year. Information will be provided on the date of inclusion of the first subject, numbers of subjects included and numbers of subjects that have completed the trial, serious adverse events/ serious adverse reactions, other problems, and amendments.

8.4 End of study report

The investigator will notify the accredited METC of the end of the study within a period of 8 weeks. The end of the study is defined as the last patient's last visit.

In case the study is ended prematurely, the investigator will notify the accredited METC, including the reasons for the premature termination.

Within one year after the end of the study, the investigator/sponsor will submit a final study report with the results of the study, including any publications/abstracts of the study, to the accredited METC.

8.5 Public disclosure and publication policy

Not applicable

9. REFERENCES

(1) Wagner LI, Cella D. Fatigue and cancer: causes, prevalence and treatment approaches. Br J Cancer 2004 Aug 31;91(5):822-8.

(2) Lucia A, Earnest C, Perez M. Cancer-related fatigue: can exercise physiology assist oncologists? Lancet Oncol 2003 Oct;4(10):616-25.

(3) Kanker in Nederland , Trends prognoses en impicaties voor zorgvraag. Signaleringscommissie Kanker van KWF Kankerbestrijding 2004.

(4) Gezondheidsraad. Nacontrole in de oncologie. Doelen onderscheiden, inhoud onderbouwen. 2007.

(5) Irwin ML, McTiernan A, Bernstein L, Gilliland FD, Baumgartner R, Baumgartner K, et al. Physical activity levels among breast cancer survivors. Med Sci Sports Exerc 2004 Sep;36(9):1484-91.

(6) National Comprehensive Cancer Network. NCCN Clinical Practive Guidelines in Oncology. Cancer-related fatigue. version 1. 2006.

(7) McNeely ML, Campbell KL, Rowe BH, Klassen TP, Mackey JR, Courneya KS. Effects of exercise on breast cancer patients and survivors: a systematic review and meta-analysis. CMAJ 2006 Jul 4;175(1):34-41.

(8) Markes M, Brockow T, Resch KL. Exercise for women receiving adjuvant therapy for breast cancer. Cochrane Database Syst Rev 2006;(4):CD005001

(9) Knols R, Aaronson NK, Uebelhart D, Fransen J, Aufdemkampe G. Physical exercise in cancer patients during and after medical treatment: a systematic review of randomized and controlled clinical trials. J Clin Oncol 2005 Jun 1;23(16):3830-42.

(10) Watson T, Mock V. Exercise as an intervention for cancer-related fatigue. Phys Ther 2004 Aug;84(8):736-43.

(11) Ahlberg K, Ekman T, Gaston-Johansson F, Mock V. Assessment and management of cancer-related fatigue in adults. Lancet 2003 Aug 23;362(9384):640-50

(12) Korstjens et al. Quality of life of cancer survivors after physical and psychosocial rehabilitation. Eur. J. of Cancer Prevention 2006, 15(6), 541-547

(13) Van Weert et al. Cancer-related fatigue: predictors and effects of rehabilitation. The Oncologist 2006: 11:184-196

(14) van Weert E., Hoekstra-Weebers JE, Grol BM, Otter R, Arendzen JH, Postema K, et al. Physical functioning and quality of life after cancer rehabilitation. Int J Rehabil Res 2004 Mar;27(1):27-35.

(15) van Weert E., Hoekstra-Weebers J, Grol B, Otter R, Arendzen HJ, Postema K, et al. A multidimensional cancer rehabilitation program for cancer survivors: effectiveness on health-related quality of life. J Psychosom Res 2005 Jun;58(6):485-96.

(16) Armitage CJ. Can the theory of planned behavior predict the maintenance of physical activity? Health Psychol 2005 May;24(3):235-45.

(17) Andrykowski MA, Beacham AO, Schmidt JE, Harper FW. Application of the theory of planned behavior to understand intentions to engage in physical and psychosocial health behaviors after cancer diagnosis. Psychooncology 2006 Sep;15(9):759-71.

(18) Courneya KS, McAuley E. Cognitive mediators of the social influence-exercise adherence relationship: a test of the theory of planned behavior. J Behav Med 1995 Oct;18(5):499-515.

(19) Ajzen I. Behavioral intervention based on the theory of planned behavior. 2006. http://www.people.umass.edu/aizen/pdf/tpb.intervention.pdf

(20) Jones LW, Courneya KS, Fairey AS, Mackey JR. Does the theory of planned behavior mediate the effects of an oncologist's recommendation to exercise in newly diagnosed breast cancer survivors? Results from a randomized controlled trial. Health Psychol 2005 Mar;24(2):189-97

(21) Zelen M. A new design for randomized clinical trials. N Engl J Med 1979 May 31;300(22):1242-5.

(22) Cardinal BJ, Esters J, Cardinal MK. Evaluation of the revised physical activity readiness questionnaire in older adults. Med Sci Sports Exerc 1996 Apr;28(4):468-72.

(23) American College of Sports Medicine Position Stand. The recommended quantity and quality of exercise for developing and maintaining cardiorespiratory and muscular fitness, and flexibility in healthy adults. Med Sci Sports Exerc 1998 Jun;30(6):975-91.

(24) Wendel-Vos GC, Schuit AJ, Saris WH, Kromhout D. Reproducibility and relative validity of the short questionnaire to assess health-enhancing physical activity. J Clin Epidemiol 2003 Dec;56(12):1163-9.

(25) Smets EM, Garssen B, Bonke B, De Haes JC. The Multidimensional Fatigue Inventory (MFI) psychometric qualities of an instrument to assess fatigue. J Psychosom Res 1995 Apr;39(3):315-25.

(26) Aaronson NK, Ahmedzai S, Bergman B, Bullinger M, Cull A, Duez NJ, et al. The European Organization for Research and Treatment of Cancer QLQ-C30: a quality-of-life instrument for use in international clinical trials in oncology. J Natl Cancer Inst 1993 Mar 3;85(5):365-76.

(27) The EuroQol Group. EuroQol – a new facility for the measurement of health-related quality of life. Health Policy 1990 16:199-207.

(28) Osborne RH, Elsworth GR, Sprangers MA, Oort FJ, Hopper JL. The value of the Hospital Anxiety and Depression Scale (HADS) for comparing women with early onset breast cancer with population-based reference women. Qual Life Res 2004 Feb;13(1):191-206.

(29) Dohoney P, Chromiak JA, Lemire D, Abadie BR, Kovacs C. Prediction of one repetition maximum (1-RM) strength from a 4-6 RM and a 7-10 RM submaximal test in healthy young adult males. Journal of Exercise Physiology online 2002;5(3):54-9.

(30) Ajzen I. Constructing a TpB questionnaire: conceptual and methodological considerations. 2002. http://www.people.umass.edu/aizen/pdf/tpb.measurement.pdf

(31) Bandura A. Social foundations of thought and action: a social cognitive theory. Englewood Cliffs, NJ: Prentice-Hall; 1986.

(32) Bassett DR, Jr., Ainsworth BE, Leggett SR, Mathien CA, Main JA, Hunter DC, et al. Accuracy of five electronic pedometers for measuring distance walked. Med Sci Sports Exerc 1996 Aug;28(8):1071-7.
